# Improving the Selectivity of the C–C Coupled Product Electrosynthesis by Using Molecularly Imprinted Polymer—An Enhanced Route from Phenol to Biphenol

**DOI:** 10.1021/acsami.3c09696

**Published:** 2023-10-12

**Authors:** Alcina Johnson Sudagar, Shuai Shao, Teresa Żołek, Dorota Maciejewska, Monika Asztemborska, Maciej Cieplak, Piyush Sindhu Sharma, Francis D’Souza, Włodzimierz Kutner, Krzysztof R. Noworyta

**Affiliations:** † Institute of Physical Chemistry, Polish Academy of Sciences, Kasprzaka 44/52, 01-224 Warsaw, Poland; ‡ Department of Chemistry, University of North Texas, 1155, Union Circle, #305070, Denton, Texas 76203-5017, United States; § Department of Organic and Physical Chemistry, Faculty of Pharmacy, Medical University of Warsaw, Banacha 1, 02-097 Warsaw, Poland; ∥ Faculty of Mathematics and Natural Sciences, School of Sciences, Cardinal Stefan Wyszynski University in Warsaw, Wóycickiego 1/3, 01-815 Warsaw, Poland

**Keywords:** molecularly imprinted polymer, electrosynthesis, homocoupling, phenol, density functional calculations, molecular dynamics

## Abstract

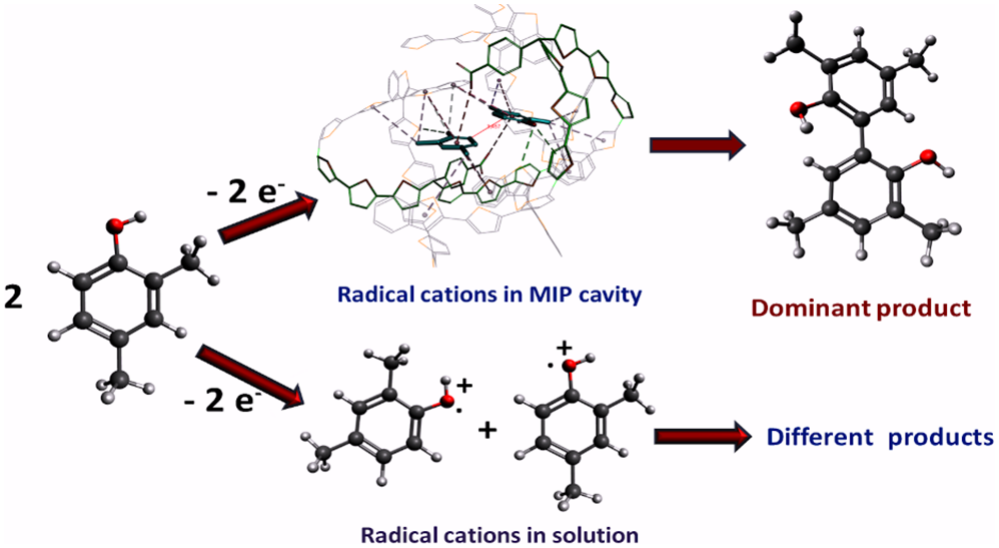

We developed a procedure for selective 2,4-dimethylphenol, **DMPh**, direct electro-oxidation to 3,3′,5,5′-tetramethyl-2,2′-biphenol, **TMBh**, a C–C coupled product. For that, we used an electrode coated with a product-selective molecularly imprinted polymer (MIP). The procedure is reasonably selective toward **TMBh** without requiring harmful additives or elevated temperatures. The **TMBh** product itself was used as a template for imprinting. We followed the template interaction with various functional monomers (FMs) using density functional theory (DFT) simulations to select optimal FM. On this basis, we used a prepolymerization complex of **TMBh** with carboxyl-containing FM at a 1:2 **TMBh-**to-FM molar ratio for MIP fabrication. The template–FM interaction was also followed by using different spectroscopic techniques. Then, we prepared the MIP on the electrode surface in the form of a thin film by the potentiodynamic electropolymerization of the chosen complex and extracted the template. Afterward, we characterized the fabricated films by using electrochemistry, FTIR spectroscopy, and AFM, elucidating their composition and morphology. Ultimately, the **DMPh** electro-oxidation was performed on the MIP film-coated electrode to obtain the desired **TMBh** product. The electrosynthesis selectivity was much higher at the electrode coated with MIP film in comparison with the reference nonimprinted polymer (NIP) film-coated or bare electrodes, reaching 39% under optimized conditions. MIP film thickness and electrosynthesis parameters significantly affected the electrosynthesis yield and selectivity. At thicker films, the yield was higher at the expense of selectivity, while the electrosynthesis potential increase enhanced the **TMBh** product yield. Computer simulations of the imprinted cavity interaction with the substrate molecule demonstrated that the MIP cavity promoted direct coupling of the substrate to form the desired **TMBh** product.

## 1 Introduction

Molecular imprinting technology aims to establish a selective binding with chosen molecules based on the “lock-and-key” mechanism known in, e.g., enzymatic reactions.^1^ When forming molecularly imprinted polymers (MIPs), functional monomers (**FMs**) are selected, which are capable of interacting with binding sites (functional groups, heteroatoms, aromatic rings, etc.) of the target compound through reversible noncovalent or covalent interactions (e.g., hydrogen bonds, the dipole–dipole, electrostatic, or van der Waals interactions). Furthermore, cross-linking monomers, **CMs**, are added to ensure the necessary polymer matrix flexibility and controlled imprinted cavities’ distribution. Since their inception,^2−8^ the MIPs have gained significant interest. Molecular imprinting is an attractive technology that fabricates MIPs exhibiting highly shape-selective interaction with target compounds, making them suitable for sensor, separation, and catalytic applications.^1^ The MIPs’ advantages over enzymes include their relatively higher stability under harsh conditions, ease of preparation and manipulation, and higher accessibility. Moreover, the enzyme system can be simplified by mimicking only its active center, thus enabling an in-depth understanding of its activity. Selective synthesis and (or) catalysis at MIPs relies on imprinting either the substrate to enhance its concentration near the reaction site or the product to promote enzyme-like shape-selective catalysis.^9^ Moreover, the reaction intermediate can be imprinted to drive the reaction through the desired route. However, the research of MIPs for selective synthesis and (or) catalysis has been limited for several reasons, including the inability to achieve selectivity as high as that provided by enzymes.^10^ This selectivity is mainly affected by the heterogeneity of the MIP cavities caused by weak intermolecular interactions. Nevertheless, the application of MIPs in catalysis has recently started to gather momentum. Some attractive MIP designs for selective catalysis of the desired products have already been described. They include the formation of MIPs with acidic recognizing sites in the imprinted cavities for selective isomerization of α-pinene oxide to trans-carveol.^11^ The Diels–Alder reaction between benzyl 1,3-butadienylcarbamate and *N*,*N*-dimethyl acrylamide, catalyzed by (methacrylic acid)-based MIPs, has also been reported.^12^ Interesting examples of amphiphilic and photoswitchable aldol reaction catalysts involving molecular imprinting have been proposed.^13^ Another example of artificial enzyme application for aldol reaction catalysis with site-isolated acid and base functionalities has been proposed.^14^ Epoxidation of alkanes using MIP-based catalysts has also been studied with exciting results.^15^ Recently, a few examples of the synthesis of enzyme-like MIP nanoparticles capable of catalyzing various reactions, including ligation of short ssDNA fragments,^16^ ester hydrolysis,^17^ and oxidation of cholesterol derivatives,^18^ have been reported. Moreover, hydrophobic cores in polymer micelles containing catalysts have been applied for molecular recognition-based catalysis.^19^ Another developing field of application of MIP-based catalysts is the selective removal of various organic compounds from industrial wastes. Removal of selected antibiotics^20^ or selective oxidation of phtalates^21^ has been successfully presented in this regard. MIPs have also been used as a support for active metal/oxide center-based catalysis, wherein the selectivity was improved through selective binding in imprinted cavities.^22−24^ Nevertheless, the application of MIPs in catalysis and selective synthesis remains underdeveloped and remains to be explored.

The oxidative coupling of phenols is an attractive synthetic route leading to primary components of natural organic products, drugs, and ligands.^25^ The homo- and cross-coupling of phenols results in several essential products of significant biological activities. 2,2′-Biphenol is a fascinating compound forming the backbone of several ligand systems applied in catalysis involving transition metals. Here, the structural motif of the ligand must be carefully selected and controlled. Specifically, the 3,3′,5,5′-tetramethyl-2,2′-biphenol, **TMBh**, is widely used in the hydroformylation reaction as a building block.^26^ Therefore, its selective synthesis is of significant practical importance. The direct oxidative homocoupling of 2,4-dimethylphenol is used for the synthesis of **TMBh**. However, the selective phenols’ oxidation to C–C coupled products is challenging because this reaction involves radical coupling and, disadvantageously, many byproducts are often formed.^27^ Therefore, it is essential to stabilize the desired intermediate that would selectively transform into the C–C coupled biphenol product. The commonly used organic syntheses of biphenols involve direct phenol oxidation with chemical oxidants or transition-metal catalysts. The reaction yields broadly vary from 12 to 98%, depending on the exact synthetic method.^28^ However, either excess oxidant, specifically designed precursor, or dedicated catalysts are needed in these cases, and the reaction leads to the production of a large quantity of harmful wastes. Moreover, the catalysts or oxidants used are often noxious and expensive.

The electro-oxidation of phenols has gained attention as a greener alternative to stoichiometric oxidative reactions as it eliminates the use of several environmentally toxic stoichiometric reagents.^29,30^ When elevated temperature and dedicated electrode materials were used, the yield and selectivity of this process have been upgraded with selectivity reaching 70%,^25^ although at the expense of the use of expensive and harmful additives or elevated temperatures. Therefore, combining MIPs providing selectivity with electrosynthesis affording controlled oxidation of phenol compounds can be a prospective solution for the above-mentioned challenges. Our work is the first example of applying MIPs for the selective electrosynthesis of desired products. It offers enormous flexibility, as the MIP can readily be imprinted with various templates to devise catalysts, selectively yielding the desired products. Artificial polymers are much more chemically and thermally stable than enzymes, and in many instances, they can match the stability of inorganic catalysts, but they are much less expensive and easier to prepare.

For the C–C phenol coupling reaction selected for our studies, applying the MIP in combination with the electrosynthesis allows for obtaining reasonable selectivity of the main biphenol product while avoiding using large quantities of harmful chemicals or expensive and unstable electrode materials, as in the existing synthetic procedures.

Herein, we have focused on the electro-oxidative homocoupling of 2,4-dimethylphenol (**DMPh**). This reaction leads to the formation of 3,3′,5,5′-tetramethyl-2,2′-biphenol (**TMBh**), a C–C coupled main product, as well as several different side products. To improve the selectivity of this coupling, we have used functionalized thiophenes for the electrochemical formation of MIP films on the electrode, imprinted with the **TMBH** reaction product, to help selectively form this desired C–C coupled product. We have selected the most appropriate FMs by DFT modeling the prepolymerization complex structure and energetics. Next, we simulated the MIP matrix structure to explain the role of the polymer in the reaction route using molecular dynamics (MD) and quantum-mechanical/molecular mechanics (QM/MM) calculations. Subsequently, the devised MIP films have been characterized, and electrosynthesis has been performed on the electrodes coated with the MIP films. The selectivity of **TMBh** product formation at the MIP film-coated electrodes has been compared to that on the bare electrodes and the electrodes coated with the reference nonimprinted polymer (NIP) film. Our research is the first example of a successful selectivity enhancement of organic electrosynthesis aided by a dedicated MIP.

## 2 Materials and Methods

### 2.1 Materials

Acetonitrile (anhydrous), dichloromethane, tetrabutylammonium perchlorate [(TBA)ClO_4_], triethylamine (TEA), and ferrocene were obtained from Sigma-Aldrich (Massachusetts) and used as received. 2,4-Dimethylphenol, **DMPh**, was procured from Merck KGaA (Darmstadt, Germany). 3,3′,5,5′-Tetramethyl-2,2′-biphenol (**TMBh**) was purchased from Strem Chemicals, Inc. (Massachusetts). The 2,2′-bis(2,2′-bithiophene-5-yl)-3,3′-bithianaphthene cross-linker (**CM**) was synthesized by the previously reported method.^31^ The compounds’ structural formulas are shown in Table S1 in the Supporting Information. Diphenylamine-2-carboxylic acid, **DACA**, was purchased from Merck KGaA (Darmstadt, Germany). The *p*-bis(2,2′;5′,2″-terthien-5′-yl) methylbenzoic acid, **BTMA**, used as an FM, was synthesized in our laboratory, and the procedure is described herewith.

### 2.2 Instrumentation

Detailed instrumentation description is given in Section S2 in the Supporting Information. Here, only the most crucial information is provided. All electrochemical experiments were performed using a Bio-Logic SAS SP-300 potentiostat/galvanostat electrochemistry system with dedicated software, using a three-electrode system. To record the UV–vis spectra, a Shimadzu UV-2501 spectrophotometer was employed. The FTIR spectroscopy measurements were performed using a Bruker Vertex 80v spectrophotometer in various configurations depending on the specific experiments’ needs. To analyze all IR spectra, the dedicated software OPUS 7 of Bruker was employed.

The ^13^C and ^1^H nuclear magnetic resonance (NMR) spectra of FMs were recorded by using an Agilent DD2 400 MH spectrometer.

Bruker’s MultiMode 8 atomic force microscope (AFM), equipped with a Nanoscope V controller, has been used for film imaging and studies of their nanomechanical properties. Either tapping or Peakforce Quantitative Nanomechanical Mapping (PF-QNM) mode was used for that purpose.

The electrosynthesis products were analyzed using an analytical high-performance liquid chromatography (HPLC) apparatus of Shimadzu Corp. (Kyoto, Japan) composed of a DGU-20A degassing unit, LC-20AT liquid chromatograph, and UV–vis diode array detector SPD-M20A. This system also contained the SIL-20A autosampler and an FRC-10A fraction collector of the same manufacturer.

A Synapt G2-S mass spectrometer (Waters, Milford, MS, USA) was used for all MS studies. It was equipped with an atmospheric pressure chemical ionization (APCI) system and a mass analyzer capable of quadrupole time-of-flight (qTOF) analysis. The MassLynx V4.1 software package (Waters) was used for the instrument control as well as for the data analyses.

### 2.3 Methods

#### 2.3.1 Computational Simulations

A series of quantum chemistry calculations were completed to study the formation of the prepolymerization complexes and select appropriate FMs. Within those calculations, optimization of the structures of the complexes and their components was performed. Moreover, the molecules’ vibrations and the changes in the Gibbs free energy accompanying the complex formation were calculated. A workstation with four Intel dual-core processors was employed for the calculations with Gaussian 09 software (Gaussian, Inc., Connecticut).^32^ The hybrid B3LYP functional together with the 6-31G(d) basis set was applied to obtain the prepolymerization complex structure of the **TMBh** template with various **FMs**, including **BTMA** and **DACA**. The calculations were performed for molecules in a vacuum at room temperature (298.15 K). Avogadro software was applied to devise the input files.^33^ The calculations allowed the obtaining of the standard Gibbs free energy changes, Δ*G*_bind_^0^, accompanying complex formation. The results helped to choose the FM most appropriate for electrosynthesis.

The simulation of the MIP cavity structure based on the experimental data for the reactants and their molar relationships was also attempted. The goal here was to model the selective electrosynthesis. First, three-dimensional structures of molecules were needed to create an MIP cavity. The **TMBh** template, the **DMPh** substrate, the deprotonated **BTMA**, and the **CM**, were designed with the help of the Discovery Studio 2020 visual interface of BIOVIA.^34^ The obtained structures underwent optimization using the DFT method implemented in Gaussian 16 software.^35^ The B3LYP hybrid functional and 6-311G(d,p) basis function were used. In agreement with the experiments, the electric permittivity (*ε*) value of the ACN/DCM (9:1, *v*/*v*) mixed solvents at 298.15 K were used for all calculations. The ε value of 34.64 *r*_ij_(36) was calculated on the basis of the experimental solvent ratios. Charges were adjusted to 0 on **TMBh**, **DMPh**, and **CM** and to −1 on the **BTMA**. To reproduce the molecular electrostatic potential, the Breneman model was used. That allowed us to calculate the electrostatic potentials from atomic partial charges.^37^ These data were used to form the cavity imprinted in the MIP.

Then, the CHARMm force field^38^ from the Discovery Studio 2020 module was used to optimize the constructed systems. First, the Gibbs free energy was minimized to relax the system (steepest descend algorithm and then the conjugate gradient algorithm with 3000 steps each). The MIP cavity was constructed using a two-step procedure to gain insight into biphenols’ synthesis. To construct the prepolymerization complex structure 1:2 molar ratio of the **TMBh** template to **BTMA** monomer was used on the basis of the ratio employed in the synthesis. First, eight **BTMA** molecules were placed around one **TMBh** molecule. The **BTMA** monomer molecules were randomly located around **TMBh**, and then the system’s energy was minimized. Subsequently, the prepolymerization complex with the 1:2 molar ratio was obtained by removing six **BTMA** monomer molecules most weakly interacting via hydrogen and π–π interactions with **TMBh**. The complex structure was then optimized again. To block the geometry of the obtained complex, a supporting potential restraining was employed. A force constant of 83.74 kJ mol^–1^ Å^2^ was used for the calculations and proved sufficient. In the following step, ten molecules of the **CM** were randomly added to the system. Then, the resulting system structure was optimized again. Next, five **CM** molecules were selected to form the prepolymerization complex with the **TMBh/BTMA**/**CM** ratio of 1:2:5 on the basis of the experimental data. Once more, the five **CM** molecules were selected on the basis of the strength of their interactions with the previously optimized **TMBh**-**BTMA** complex. Finally, the structure of the generated complex was optimized one another time. This final structure was stabilized by simulating electropolymerization, leading to a theoretical model of the recognizing cavity. Previous results of modeling electropolymerization for similar systems^39^ suggest that covalent bonds would form between the C2 atoms of thiophene rings of neighboring **BTMA** and **CM** molecules. Thus, the formed cavity structure was optimized. Then, in the last step, molecular dynamics (MD) was used to generate the final cavity structure. The adopted procedure for MD calculations was as follows: (i) the systems were heated from 0 to 298.15 K within 50 ps (1 fs per step) and then equilibrated thermally for 100 ps (1 fs per step), which allowed the equilibration state. This system was then used as the starting structure for 5 ns production runs with the canonical ensemble (NVT, 298.15 K) with the use of a Berendsen thermostat.^40^ The leapfrog Verlet algorithm and Langevin temperature coupling method were used here. To freeze the 3D structure of the imprinted cavity during MD simulations, the constraints were applied to heavy atoms with a force constant of 418.68 kJ mol^–1^ Å^2^. Then, the template molecule was removed from the system. The MIP cavity obtained that way was used as the theoretical model of the active site used for the biphenols synthesis.

To simulate the electro-oxidation of **DMPh** to the **TMBh** C–C coupled product in the molecular cavity, the combined quantum-mechanical/molecular mechanical (QM/MM) methods were applied. A two-layer ONIOM scheme implemented in the Gaussian 16 software package was employed to perform the QM/MM calculations. This method is based on a hybrid quantum chemical approach proposed by Morokuma et al.,^41,42^ where different levels of theory are used for different systems’ parts. The systems’ most essential components are calculated by using a high-level QM method, which can adequately describe chemical bond forming and breaking. Besides, a low-level computational method (often MM) is used to calculate less critical parts of the system. The influence of the molecular surroundings on the tested system can be correctly described using this approach. In our work, the hybrid functional B3LYP^43−45^ was used as the high-level method, while the low-level method employed was the Amber force field.^46^ The correct applicability of the ONIOM (B3LYP: Amber) method has already been shown for several enzymatic systems.^47^ In our simulations, the QM region encompassed the **TMBh** product or the **DMPh**^**•+**^ radical cations acting as substrates. In order to saturate the reaction, two hydrogen ions were used. In all calculations, the 6-311G basis set was applied for optimization of molecules’ geometry. The outlying parts of the system, that is, the imprinted cavity, were considered the MM region and were treated by the Amber force field. The SCRF-SMD solvation model was adopted with an electric permittivity ε = 34.64 to simulate the reaction environment in the QM cluster calculations. The electrostatic embedding method of Gaussian 16 was applied to estimate the Coulomb interactions between MM and QM regions in all ONIOM calculations.^48^ Starting systems for QM/MM calculations were constructed by the consecutive insertion into the cavity model of two molecules of the **DMPh** and their radical cations **DMPh**^**•+**^ as substrates, as well as the molecule of **TMBh** and 2H^+^ as products. Those systems were again MD simulated and finally optimized. The standard Gibbs free energy changes of the reactants in the polymer matrix were taken into account when the calculations of the standard Gibbs free energy changes of the studied reactions were performed.

#### 2.3.2 *p*-Bis(2,2′;5′,2″-terthien-5′-yl) Methylbenzoic Acid (**BTMA**) Functional Monomer Preparation

Preparation of *p*-bis(2,2′;5′,2″-terthien-5′-yl) methylbenzoic acid, **BTMA**, monomer is described and presented in Section S1 and Scheme S1 in the Supporting Information, respectively.

#### 2.3.3 Fabrication of the Molecularly Imprinted Polymer (**MIP**) Film, as well as the Reference Nonimprinted Polymer (**NIP**) Film

Cyclic voltammetry (CV) and differential pulse voltammetry (DPV) studies were completed at room temperature [25 ± 1 °C] in an electrochemical V-shaped three-neck glass minicell (Scheme S2 in the Supporting Information). Before MIP film deposition, sandpaper (PP 2500) was used to roughen the Pt working electrodes. The electrodes were then cleaned with deionized water and acetone in an ultrasonic bath for 15 min. The goal was to improve the adhesion of the polymer film to the electrode surface. The **MIP-a** films were fabricated by oxidative potentiodynamic electropolymerization. For that, five potential cycles were applied in the range from 0 to 1.40 V vs Ag quasi-reference electrode at a scan rate of 50 mV s^–1^. The 0.1 M tetrabutylammonium perchlorate [(TBA)ClO_4_] in ACN/DCM (9:1, v/v) solution containing 200 μM**TMBh** template, 400 μM **BTMA**, 1000 μM in 2,2′-bis(2,2′-bithiophene-5-yl)-3,3′-bithianaphthene **CM**, and 1000 μM TEA was used for polymerization. The **CM** synthesis was reported previously.^31^ A thinner MIP film, **MIP-b**, was prepared using a solution of prepolymerization complex diluted ten times with 0.1 mM (TBA)ClO_4_ in ACN/DCM (9:1, v/v).

In order to extract the template from the MIP film after electropolymerization, the (acetic acid)/methanol (1:1, v/v) solution was employed. The electrode coated with the MIP film was immersed for up to 180 min in the above-mentioned solution at room temperature, 25 (±1) °C. To follow the extraction procedure, a DPV curve in the presence of the ferrocene redox probe has been recorded using MIP film-coated electrodes as the working electrodes. The 100 mM (TBA)ClO_4_ in ACN/DCM (9:1, v/v) solution was used as the supporting electrolyte. The potential was recorded in the 0.00–0.80 V range vs Ag quasi-reference electrode. A potential step of 5 mV and a 2.5 mV pulse amplitude of 100 ms duration was used. The reference **NIP-a** and **NIP-b** films were synthesized similarly to analogous MIP films by oxidative electropolymerization but without the **TMBh** template. The NIP films were also immersed in the same solution used for MIP extraction to maintain treatment conditions similar to those experienced by MIP films.

#### 2.3.4 Electrochemical Synthesis of 3,3′,5,5′-Tetramethyl-2,2′-biphenol, **TMBh**

**TMBh** was electrosynthesized under potentiostatic conditions by **DMPh** electro-oxidation. A 0.10 M (TBA)ClO_4_ ACN/DCM (9:1, v/v) solution and room temperature [25 (±1) °C] were employed in all syntheses. The Bio-Logic SP-300 potentiostat and the three-electrode glass minicell (Scheme S2 in the Supporting Information) were used. Smaller Pt disk working electrodes, 0.75 mm in diameter and area of 0.44 mm^2^, were applied for preliminary synthesis studies. For large-scale electrosyntheses, larger Pt electrodes, viz., plates of 22 × 5 mm^2^ (2.74 cm^2^ area) were used. The active surface area of the larger electrode was 1.90 cm^2^. A Pt wire or a 4 mm diameter 45 mm long graphite rod was applied as the counter electrode for smaller or larger working electrodes, respectively. A silver wire 1 mm in diameter was used as the quasi-reference electrode. The electrode’s potential was referred to that of the ferrocene redox process and used as the internal reference of potentials. The solution was not stirred during the electrosynthesis. Therefore, the reaction substrates and products diffused into and out of the polymer films. Before electrochemical synthesis, the ten potential cycles from 0.00 to 2.00 V vs Ag quasi-reference electrode were performed for both the MIP and NIP films in the 100 mM (TBA)ClO_4_ in the ACN/DCM (9:1, v/v) solution. That allowed for the preparation of films of low conductivity to avoid parasitic reactions. The MIP film thickness, electrosynthesis potential and time, and the **TMBh** template concentration effect on the primary product yield and conversion were analyzed. Time dependence of the synthesis parameters was studied for electrosyntheses at the bare, MIP, or NIP film-coated electrodes to understand the superior performance of the MIP film in the selective electrosynthesis of **TMBh**. The substrate conversion and product yield were calculated using the HPLC calibration plots, considering the stoichiometry of the phenol coupling. The electrosynthesis selectivity was calculated using eq 1, assuming that the total product concentration equals the amount of substrate converted.
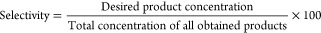
1

## 3 Results and Discussion

### 3.1 Functional Monomers Choice

Devising an MIP for selective synthesis requires imprinting of the template resembling the structure of the desired reaction transition state. That is often challenging, as the transition state is usually unstable and its structure may be unknown. Therefore, this approach requires first finding the desired transition state structure and then finding an analogue closely resembling it. Thus, the approach based on the desired reaction product is often used instead.^13^ This approach can be pretty successful and facile, provided that the transition state structure is similar to that of the desired product. In our case, biphenol synthesis is based on coupling two phenol radicals. Hence, the structure of the expected transition state leading to the final desired product is quite similar to the final product structure. Therefore, the **TMBh** product was chosen as the template for molecular imprinting.

Apparently, oxygen-containing functional groups capable of proton accepting and donating (carboxylic and boronic acid groups) offered stronger **TMBh** template binding. Furthermore, **DACA** and **BTMA**, containing carboxyl groups, strongly interacted with **TMBh** electrostatically if these groups were deprotonated.

**Scheme 1:**
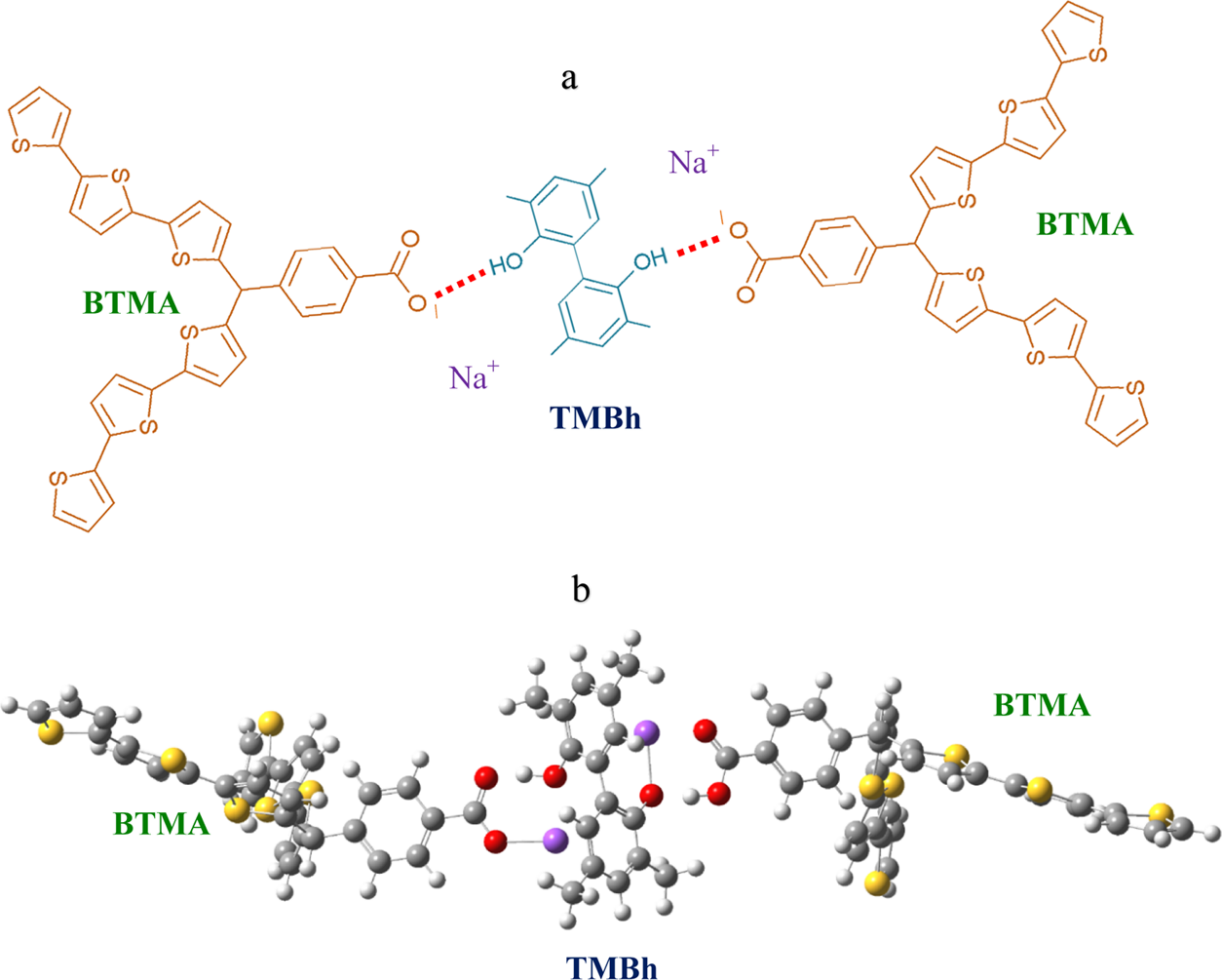
Prepolymerization Complex: (a) Structural Formula and (b) the DFT B3LYP/6-31g(d) Optimized Structure in a Vacuum of the **TMBh** Template with the Deprotonated Form of **BTMA** at the Ratio of 1:2 (mol/mol) Na^+^ ions were used for charge neutralization.

Successful preparation of MIP films requires strong interactions between the **TMBh** template and **FM**. The **DACA** strongly bound the **TMBh** template. Unfortunately, **DACA**-**TMBh** prepolymerization complex electropolymerization appeared difficult, forming a relatively thin and compact film (Figure S1 in the Supporting Information). Moreover, the template extraction from this film was unsuccessful, possibly because of the formation of such a thin, compact film impermeable to the extracting solution. Therefore, the **BTMA** was chosen because of its respectable binding with the **TMBh** template, especially at the 1:2 molar ratio. The Δ*G*_bind_^0^ accompanying the interaction between the template and the deprotonated form of **BTMA** was −68 kJ mol^–1^ (Scheme 1 and Table S2 in the Supporting Information). FM deprotonating was vital for generating strong interactions. Notably, the formation of strong hydrogen bonds in the **TMBh**-**BTMA** complex results in a substantial proton shift from the −OH moiety of **TMBh** to the ^–^OOC– moiety of **BTMA**. Moreover, the chosen **FM** polymerizes efficiently.

UV–vis and FTIR spectroscopy studies (Figures S2 and S3 in the Supporting Information) confirmed the influence of interactions between the **TMBh** template and **BTMA** on individual molecules’ electronic transitions and vibrations. The **BTMA** π–π* transitions manifested as bands between 300 and 400 nm are relatively strong, thus masking the smaller changes expected during the complexation of **TMBh** with **BTMA** (Figures S2a and S2b, respectively, in the Supporting Information). Therefore, subtle changes between experimental and simulated spectra can only be observed below 300 nm. A small difference between the simulated and experimental spectra in the absence and presence of TEA for the **TMBh**/**BTMA** mixtures is observed below 230 nm (Figures S2a and S2b, respectively, in the Supporting Information). The differences are more pronounced for the 1:2 complex of **TMBh**/**BTMA**, presumably pointing toward weak electrostatic interactions affecting the −COOH and −OH groups’ electronic transitions, respectively, in the **BTMA** and **TMBh**. Those groups are expected to interact in the prepolymerization complex. A band ascribed to the n-π* and π–π* transitions^49^ in the benzoic acid moiety of **BTMA** is expected at ∼230 nm. In this case, orbitals are partially located on the −COOH group, being affected by hydrogen bonding with the −OH group of **TMBh**. Similarly, spectra of phenols typically exhibit π–π* transitions at ∼270 nm, where changes are also observed in the presence of TEA. However, the spectral changes below 215 nm cannot be considered conclusive because of the limits of the spectrophotometer resolution. The general absorbance of **BTMA** and **TMBh**-**BTMA** below 230 nm was augmented upon TEA addition.

The FTIR studies of the prepolymerization complex formation are presented in Figures S3 and S4 (Supporting Information). The strong, broad intermolecular-bonded OH stretching bands of **BTMA** between 3400 and 3600 cm^–1^ are affected by the addition of **TMBh** (Figures S3a and S4a in the Supporting Information), leading to variations in their intensity. Furthermore, the shoulder bands at 3544 and 3481 cm^–1^ become more pronounced in the complex compared with the main band. The changes in relative intensity of bands in these regions also lead to the main band position change to 3524 cm^–1^ for complexes, compared to 3522 cm^–1^ for **TMBh**. The changes in this spectral region are more pronounced in the presence of TEA. Also, shoulder bands at 3547 and 3481 cm^–1^ become more prominent (Figures S3b and S4b in the Supporting Information). Moreover, the main band shifts to higher wavenumbers. Furthermore, the relative intensity of the band at ∼1696 cm^–1^ to that of the carboxyl C–O stretching in **BTMA** at 1718 cm^–1^ increased upon complexation, resulting in the increase of intensity of the band at 1696 cm^–1^ (Figures S3c, S 3d, S4c, and S 4d, respectively, in the Supporting Information). These relative intensity changes are more visible when complexation occurs in the presence of TEA (Figures S3d and S4d in the Supporting Information). The differences between the UV–vis and FTIR spectra for the **BTMA** and **TMBh** components and that for the 1:1 and 1:2 complexes of **TMBh** with **BTMA** suggest binding of **TMBh** with **BTMA** occurring through −OH and −COOH groups, in agreement with the DFT simulations’ predictions.

### 3.2 Fabrication of Molecularly Imprinted Polymer (**MIP**), as well as Nonimprinted Polymer (**NIP**) Films

**Scheme 2:**
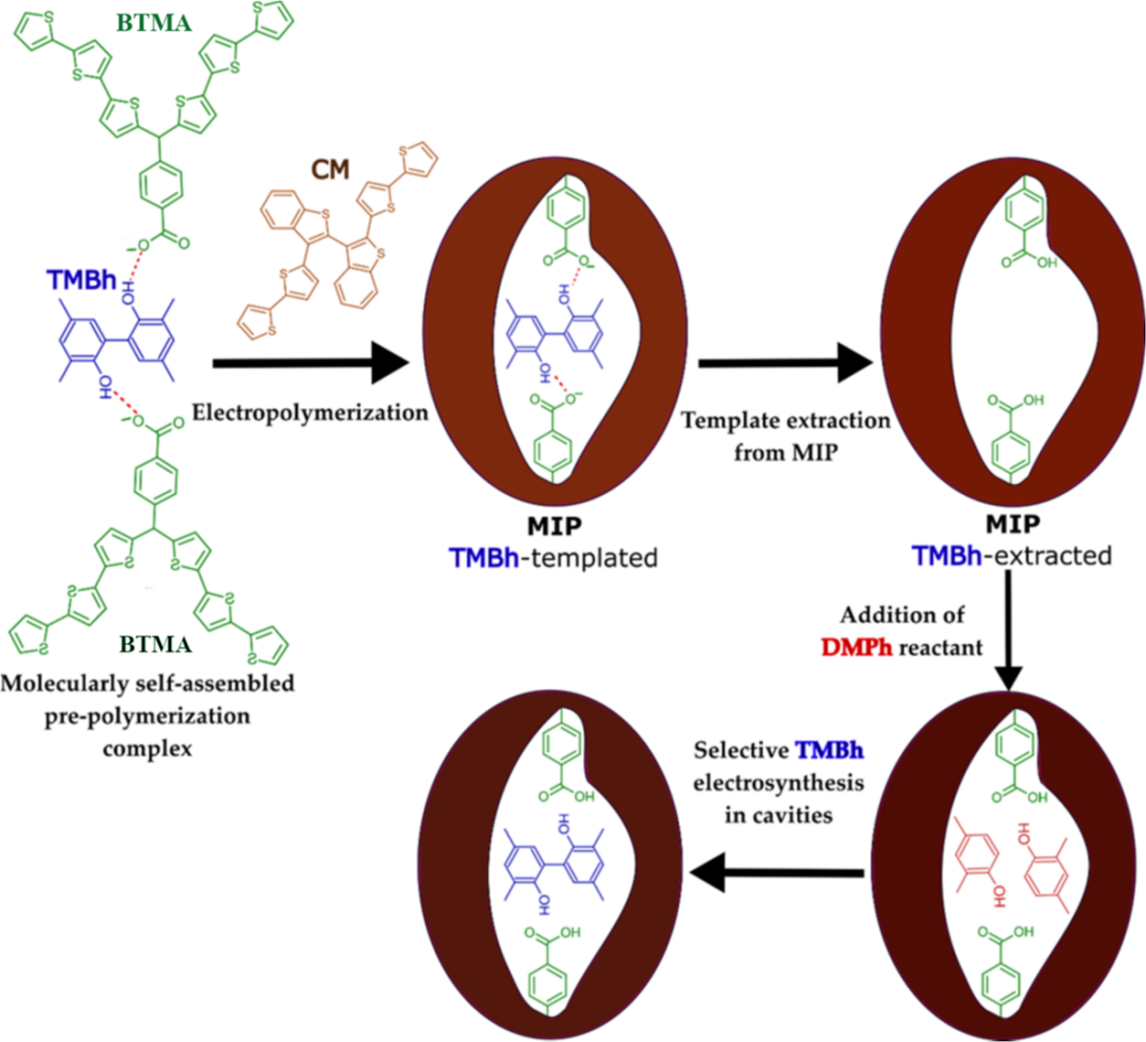
Preparation of **MIP**s with Selectivity toward **TMBh** and the Proposed **TMBh** Electrosynthesis Mechanism Inside the Film

The prepolymerization complex contained **TMBh**/**BTMA**/**CM** at the 1:2:5 molar ratio in the ACN/DCM solution of TEA and (TBA)ClO_4_. The DCM (10 vol %) enhanced the **BTMA** solubility. The **BTMA** interacted with the **TMBh** template, as DFT simulations revealed. Moreover, the **BTMA** electropolymerization at potentials lower than those of the template is significant to maintaining the integrity of the template. TEA was used to deprotonate the **BTMA** to enable a stronger **TMBh**-**BTMA** interaction. The **CM** was used as it undergoes three-dimensional polymerization, yielding a relatively open and rigid 3-D networked film. Scheme 2 shows the preparation of the electrode coated with the **MIP** film.

**Figure 1:**
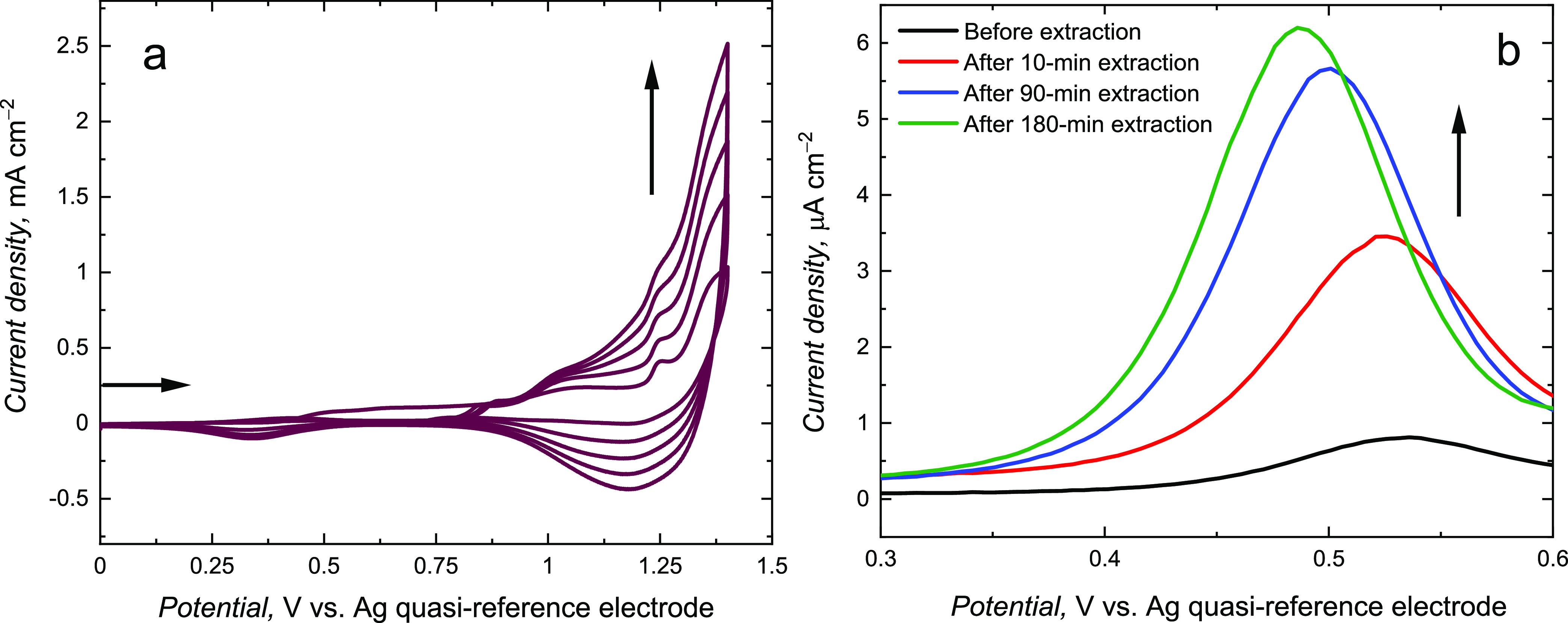
(a) Current–potential curves recorded during potentiodynamic electropolymerization of the **MIP-a** film on a Pt disk electrode in the 100 mM in (TBA)ClO_4_, ACN/DCM (9:1, v/v) solution, with 200 μM **TMBh**, 400 μM **BTMA**, 1000 μM **CM**, and 1000 μM TEA. The deposition was performed at a scan rate of 50 mV s^–1^ using five consecutive potential cycles. (b) DPV results obtained for the electrode coated with the **MIP-a** film in the 100 mM (TBA)ClO_4_, ACN/DCM solution of the 1 mM ferrocene redox probe before and after template extraction with (acetic acid)/methanol (1:1, v/v). Extraction time intervals are indicated at curves.

The **TMBh**-selective **MIP** films were formed on the electrode by potentiodynamic oxidative electropolymerization using five cycles from 0.00 to 1.40 V vs Ag quasi-reference electrode (Figure 1a). The observed current increase during each consecutive cycle indicated conductive **MIP** film deposition.

The anodic peak starting at ∼1.20 V vs Ag quasi-reference electrode indicates the formation of radical cation during the electro-oxidation of the **BTMA** and **CM**’s terthiophene and bithiophene moieties, respectively. In ensuing cycles, all anodic peaks grow in the potential range of 0.80 and 1.10 V vs Ag quasi-reference electrode. That indicates polythiophene chain formation, oxidized at potentials less positive than the bithiophene and terthiophene moieties.

**Figure 2:**
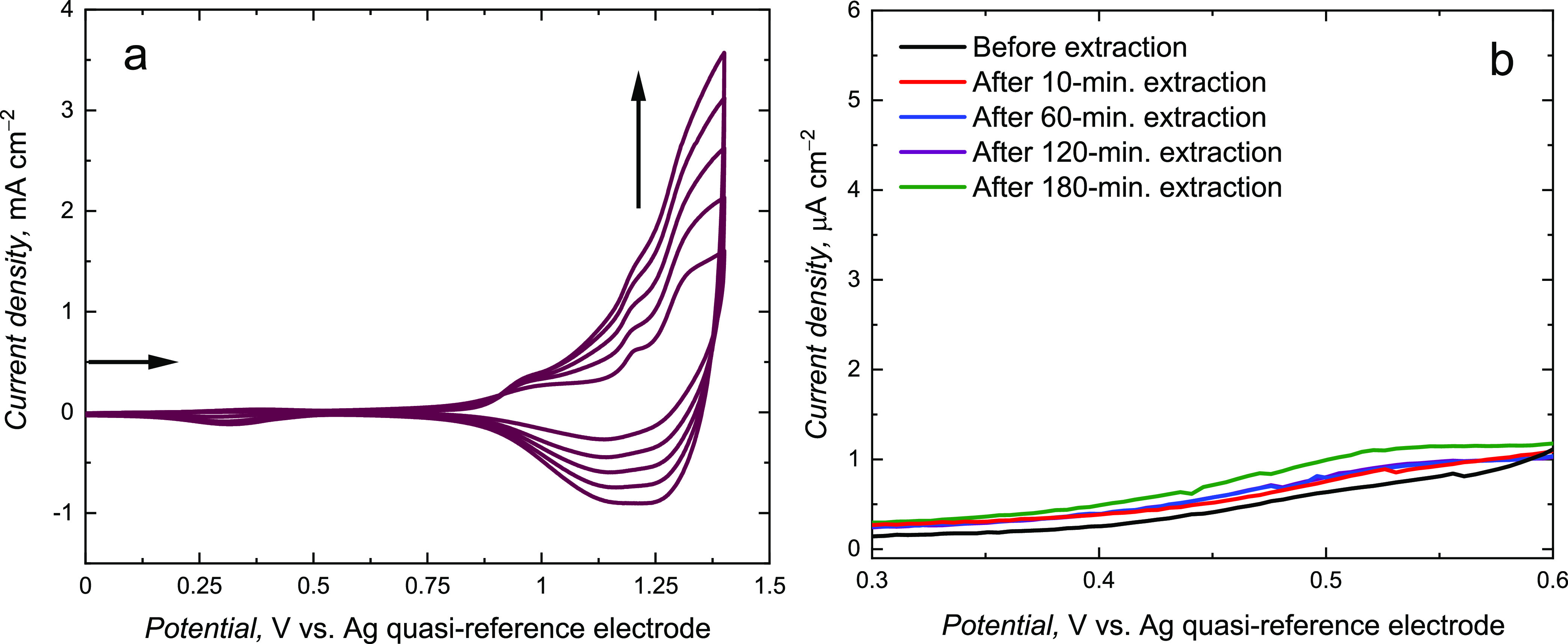
(a) Current–potential curves recorded during potentiodynamic electrodeposition of **NIP-a** film at 50 mV s^–1^ in the 100 mM (TBA)ClO_4_, acetonitrile: dichloromethane solution of 400 μM **BTMA**, 1000 μM **CM**, 1000 μM TEA. The deposition was performed with five consecutive potential cycles. (b) DPV curves for the **NIP-a** film in the 100 mM (TBA)ClO_4_ acetonitrile:dichloromethane solution of 1 mM ferrocene. Curves were recorded before and after immersion of **NIP-a** in the (acetic acid)/methanol (1:1, v/v) solution.

The CV curve observed during **NIP** film electrodeposition was similar to that for **MIP** film deposition (Figure 2a), although currents during **NIP** deposition exceeded those for MIP deposition. This effect indicates the influence of the formed prepolymerization complex on the electrodeposition process. Then, the template molecules were removed from cavities in the **MIP** film using extraction with (acetic acid)/methanol (1:1, v/v), thus vacating molecular cavities. Acetic acid interaction with the template is stronger than that with the **FM**. Therefore, this allows for its removal from the **MIP** film.

The extraction was followed by DPV with the “gate effect” by applying ferrocene as the redox probe (Figure 1b).^50^ The **MIP** film initially blocked the redox probe’s access to the surface of the electrode; therefore, the DPV peak for ferrocene oxidation is small. After template extraction, this peak increased. The observed peak potential shift presumably resulted from lowering the ohmic potential drop because of facilitated redox probe transfer through the film. After 180 min extraction, the DPV peak current was the highest. The DPV peak did not rise for longer time intervals, suggesting complete extraction.

The **NIP** films were immersed in the same solution and under the same conditions as those used for **MIP** films’ extraction (Figure 2). The **NIP-a** films were also conductive (Figure 2a). However, the DPV peak for the redox probe did not rise much despite the fact that the **NIP-a** film-coated electrode was treated with the (acetic acid)/methanol (1:1, v/v) solution for 180 min (Figure 2b). Notably, the DPV peak for the fully extracted electrode coated with MIP film was ∼9 times higher than that for the extracting solution-treated NIP film, indicating the effective formation of imprinted cavities.

### 3.3 Characterization of **MIP** Film-Coated Electrodes

FTIR spectroscopy was used to characterize **MIPs** and **NIPs** after films’ deposition and after **TMBh** template extraction from the MIP film and immersion in the solution for extraction of the **NIP** film. Two sources of spectral differences may be identified, i.e., **MIP** and **NIP** film composition differences and the changes after film treatment with the solution for extraction (Figure S5a in the Supporting Information).

The bands between 2800 and 3000 cm^–1^, corresponding to the −CH stretching vibrations (Region 1 in Figure S5a in the Supporting Information), differ significantly for thinner **MIP-b** and **NIP-b** films. The bands for the **MIP-b** films are more intense than those for the **NIP-b** films. Intensities of those bands clearly decrease after both films’ exposure to the (acetic acid)/methanol (1:1, v/v) extracting solution. Additionally, between 3000 and 3500 cm^–1^ new bands can be observed linked with −OH stretching vibrations in protonated carboxyl moieties. Moreover, spectral differences between **MIP-b** and **NIP-b** films are manifested in the 1600–1800 cm^–1^ region (Region 2, Figure S5a in the Supporting Information). Here, two signals at 1760 and 1733 cm^–1^ and a weak broad peak at ∼1655 cm^–1^ are visible for the nonextracted **MIP-b**. Those bands may be assigned to vibrations of the C=O group interacting with the template. In the extracted **MIP-b** film, the band at 1733 cm^–1^ shifted to ∼1722 cm^–1^, and its intensity decreased. The band at 1760 cm^–1^ completely vanished. However, the band at ∼1655 cm^–1^ remained visible. These spectral changes indicate a variation in the C=O group interaction, supporting the removal of the template. The signals at 1722 cm^–1^ and 1655 cm^–1^ were present for **NIP-b** before immersion in the extraction solution. Those bands did not change after **NIP-b** immersion in the solution used for extraction. Regions 3 and 4 in Figure S5a in the Supporting Information disclose the extraction effect on the films by the appearance of two major signals at 1430 and 1120 cm^–1^. The band at 1430 cm^–1^ is typically connected with either −C–H or −OH bending, while the band at 1120 cm^–1^ is located in the C–O stretching region. These two bands indicate the (extracting solution)-incurred film structure changes and may be associated with protonation of the carboxylate during immersion in the solution for extraction. We used two spectroscopy techniques for film analysis: grazing angle FTIR and PM-IRRAS. Both techniques are surface-sensitive and can measure thin film IR spectra. PM-IRRAS is additionally sensitive to the orientation of molecules in the film, thus allowing for insight into the film organization. Interestingly, there were significant differences in the thicker **MIP-a** film’s PM-IRRAS and grazing angle FTIR spectra (Figure S5b in the Supporting Information). Different relative intensities of the bands in the four regions (marked with red dotted lines) can be distinguished in the PM-IRRAS and grazing angle FTIR spectra. The bands between 2800 and 3100 cm^–1^ decreased importantly. Similarly, the bands at ∼1350 and ∼1105 cm^–1^ also decreased. Besides, the signal at ∼735 cm^–1^ increased compared to its neighbors in PM-IRRAS spectra. These changes can be explained as indicative of the oriented growth of the polymer film. In PM-IRRAS spectra, the intensity of vibrations with in-plane transition dipole moments decreased, while the intensity of signals linked to vibrations with transition dipole moments oriented out-of-the-plane increased. Therefore, the changes in the relative intensities of the mentioned bands point out local ordering of the molecules in the film. Such organization was not evident in the thinner **MIP-b** film, as grazing angle FTIR spectroscopy was insufficiently sensitive.

The **MIP-a**, **NIP-a**, **MIP-b**, and **NIP-b** films were imaged with AFM before and after immersion in the (acetic acid)/methanol (1:1, v/v) solution. The morphology and nanomechanical properties of the**MIP** and **NIP** films were investigated. Both films were relatively rough, with large thickness (Figures S6a and S6c, respectively, and Table S3 in the Supporting Information). The average film thickness for **MIP-a** and **NIP-a** films was in the range of 657–883 nm. Both films showed similar roughness, exceeding 200 nm, and were composed of granular aggregates, which formed layered structures. The **MIP-a** and **NIP-a** films’ structures were changed only slightly after immersion in the extracting solution (Figures S6b and S6d, respectively, in the Supporting Information). However, the thickness and roughness of the **MIP-a** film increased after extraction, most probably indicating swelling of the film during this process (Table S3 in the Supporting Information). As shown in Figure S7 and Table S3 in the Supporting Information, the films deposited from a 10-times diluted solution (**MIP-b** and **NIP-b** films) were noticeably thinner and less rough. The **MIP-b** film’s average thickness reached 488 (±18) nm, and its roughness was 45 (±14) nm. The **NIP-b** film’s average thickness was relatively lower, reaching 384 (±31) nm, and its roughness was smaller (∼20 nm). Both films were compact and homogeneous, with clusters of aggregates on top (Figures S7a and S7c, respectively, in the Supporting Information). Immersion of **MIP-b** and **NIP-b** films in the extracting solution may lead to film swelling, but this effect is more than offset by the removal of top aggregates (Figures S7b and S7d, respectively, in the Supporting Information), leading to the net decrease of the average thickness of **MIP-b** (Figure S7b in the Supporting Information). The mapping of the Young modulus for **MIP** and **NIP** films (Figure S8 in the Supporting Information) points out that the films are composed of softer and harder regions. The film’s average Young modulus was in the range 1–7 GPa (Table S3 in the Supporting Information). The Young modulus values for polythiophene films are typically reported as close to a few GPa.^51,52^ The average Young modulus values were higher in the case of **NIP** than in the case of **MIP** for all films studied. That indicates that softer films were formed during the imprinting.

### 3.4 Electrochemical Synthesis of **TMBh**

The cyclic voltammogram for **DMPh** recorded in the (TBA)ClO_4_-containing ACN solution showed an anodic peak at 1.00 V vs Ag quasi-reference electrode. This peak is associated with **DMPh** electrochemical oxidation (Figure S9 in the Supporting Information). Therefore, the potentiostatic electrochemical synthesis must be performed above 1.00 V vs Ag quasi-reference electrode to yield the desired C–C coupled **TMBh** product. The **DMPh** anodic oxidation resulted in a radical cation acting as a strong Brønsted acid. Being unstable, this acid then expels a proton, forming a phenoxyl radical. This radical has spin densities distributed over many sites. Therefore, this radical can subsequently be converted to the phenoxonium species. These phenoxonium and phenoxyl species can attack the **DMPh** molecule or other nucleophile molecules that were produced during electro-oxidation, consequently leading to bond formation.^53^ The carbon–carbon coupling occurs via the unsubstituted reactive site of the substrate. However, due to the presence of multiple reactive sites on the intermediate unstable molecules, numerous side products are probable besides the desired **TMBh** product.

Before electrosynthesis, conditioning **MIP** and **NIP** films was performed by applying ten current–potential cycles in the range of 0.00–2.00 V vs Ag quasi-reference electrode in the ACN/DCM (9:1, v/v) solution of 0.1 M (TBA)ClO_4_ (Figure S10 in the Supporting Information). The current decreased significantly after the first cycle, indicating that the film became less conductive. The film conductivity decreased further with each subsequent cycle. This process was introduced to the MIP preparation procedure to reduce the side reaction between the oxidized **DMPh** substrate and the film during electro-oxidation. This effect has been observed for a nonconditioned **MIP** film. After film conditioning, **DMPh** electro-oxidation was carried out. The electro-oxidation of **DMPh** to obtain **TMBh** was executed under potentiostatic conditions at the **MIP-a**, **MIP-b**, and **NIP-a** film-coated, as well as at noncoated Pt plate working electrodes (active surface area of ∼1.90 cm^2^). The AFM imaging indicated that the films were very rough, facilitating substrate diffusion to the imprinted cavities as well as diffusion of the obtained product out of the film. This diffusion is also facilitated by not overly strong binding of the substrate and product within the cavities, as indicated by the quantum-mechanical calculations.

The charge passing through the **MIP** and **NIP** film-coated and bare electrodes during electrochemical syntheses was measured (Figure S11 in the Supporting Information). Shapes of the charge evolution with time were different for the electrodes coated with film compared to those recorded for the bare electrode. The charge increased slowly during the initial 2 h in the case of both **MIP** and **NIP** film-coated electrodes and then rose faster before finally tending toward a plateau. The initial slow charge increase was much less prominent for the bare electrode. This effect could be indicative of initial intermediate product accumulation and then faster electro-oxidation to the final product. The charge passed during electrosynthesis on the **NIP-a** film-coated electrode was the lowest. It reached a plateau after ∼3 h, beyond which the charge rose very slowly (Figure S11 in the Supporting Information). The charges measured for the bare and **MIP-a** film-coated electrodes were similar, but the charge measured for the electrode coated with the **MIP-a** film reached a plateau after ∼10 h. Charges passed on electrodes coated with thinner **MIP-b** and **NIP-b** films were lower than those measured for thicker **MIP-a** and higher than those measured for **NIP-a**. However, the thinner films’ transients and curve shapes were like those of thicker films. Several samples of the reaction mixture were collected at different synthesis times and analyzed by the HPLC.

**Figure 3:**
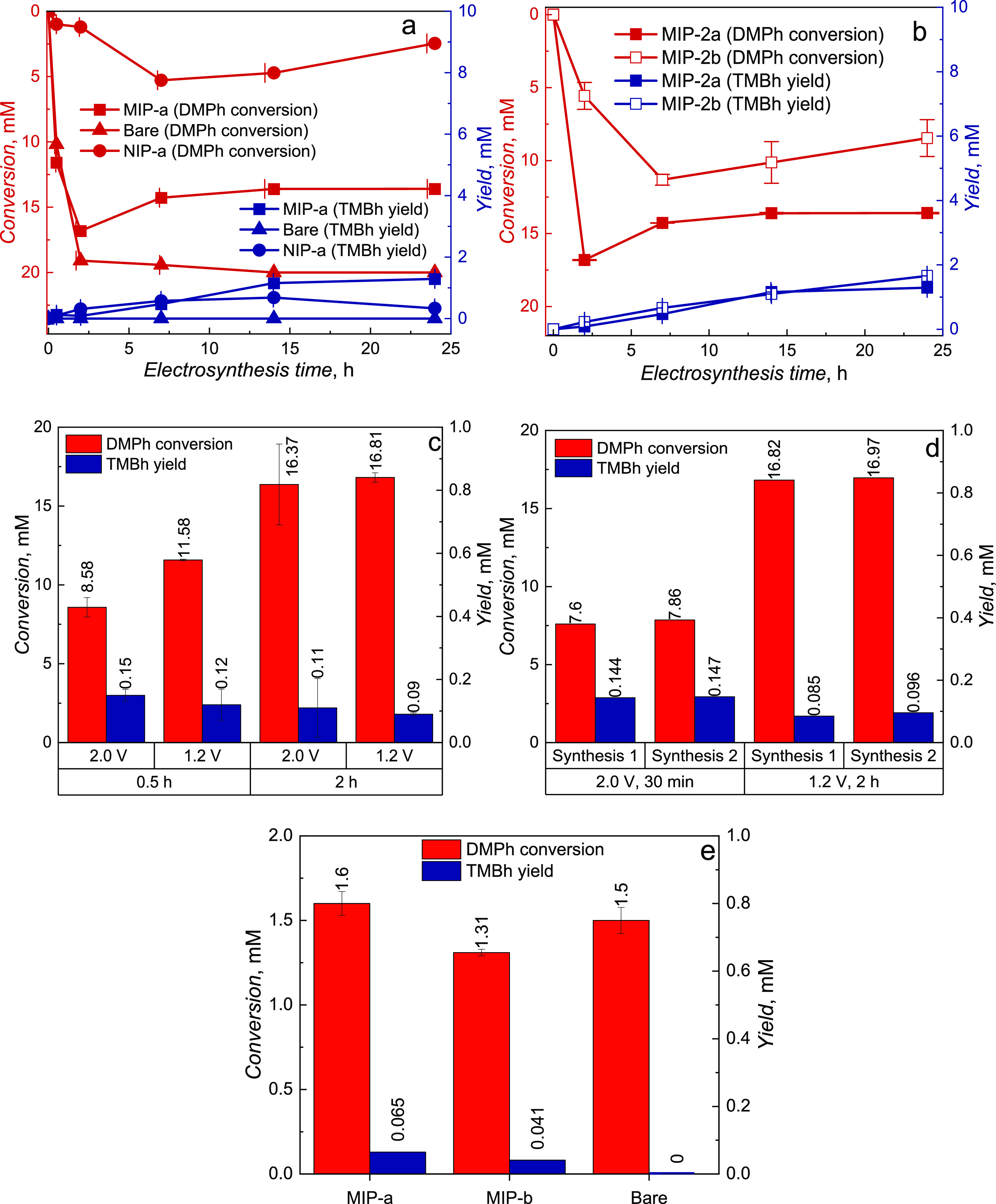
Conversion of the **DMPh** substrate and the **TMBh** product yield obtained for different synthesis times for (a) the noncoated Pt electrode and Pt electrodes coated with thicker **MIP-a** and **NIP-a** films, as well as for (b) thinner **MIP-a** and **MIP-b** film-coated electrodes. The reactions were performed in 20 mM **DMPh** in 0.1 M (TBA)ClO_4_, ACN/DCM (9:1, v/v) supporting electrolyte solution. The applied constant potential was 1.20 V vs Ag quasi-reference electrode. Electrosynthesis was performed at the electrodes coated with thicker **MIP-a** (c) at various electrode potentials and (d) during various numbers of synthetic runs. Syntheses were performed in a 20 mM **DMPh** in 0.1 M (TBA)ClO_4_, ACN/DCM (9:1, v/v) supporting electrolyte solution. The applied constant potential was 1.20 V vs Ag quasi-reference electrode. (e) Electrosynthesis yield and conversion at noncoated, as well as at electrodes coated with **MIP-a** and **MIP-b** films. Electrosynthesis was performed in 2 mM **DMPh** in 0.1 M (TBA)ClO_4_, ACN/DCM (9:1, v/v) supporting electrolyte solution under potentiostatic conditions at 1.20 V vs Ag quasi-reference electrode during 7 h.

The HPLC chromatograms and peak area calibration plots for the substrate and desired product are presented in Figure S12 (Supporting Information). Typical chromatograms recorded for solutions after the electrosynthesis are presented in Figure S13 in the Supporting Information. The **DMPh** substrate conversion and **TMBh** yield at the bare and **MIP** and **NIP** film-coated electrodes versus synthesis time are shown in Figure 3. The potentiostatic electrochemical oxidation performed at 1.20 V vs Ag quasi-reference electrode resulted in ∼75% **DMPh** substrate conversion after 24-h electrosynthesis at the **MIP-a** film-coated electrode, while the conversion was significantly lower at the electrode coated with the **NIP-a** film, not exceeding 40% (Figure 3a). The electrode coated with the **MIP-a** film electrode exhibited the highest **TMBh** product yield after 14 h of synthesis, after which the yield increased only slightly. Oppositely, **DMPh** practically entirely vanished after 14 h of electrosynthesis at the bare Pt electrode, although the HPLC analysis of the reaction mixture revealed only a minuscule presence of the desired **TMBh** product (Figure S13 in the Supporting Information). Apparently, electrosynthesis at the noncoated electrode does not result in the formation of the **TMBh** product.

The **NIP-a** film appears to block the electrode, resulting in very low substrate conversion and product yield. Moreover, a higher **DMPh** conversion was observed for the **MIP-a** film-coated electrode. However, the **TMBh** yield at this thicker **MIP-a** film was somewhat lower than that at the thinner **MIP-b** film (Figure 3b).

Ergo, the electrosynthesis selectivity for the **TMBh** desired product of thinner **MIP-b** films was higher, reaching up to ∼39% after 24 h synthesis time, as determined based on the conversion measured. The selectivity of the syntheses described in the literature is compiled in Table 1 for comparison.

**Table 1:** Selectivity of Various Methods of DMPh Biphenol Electrosynthesis Described in the Literature

conditions	selectivity, %	ref
aqueous–organic solvents at room temperature	17	(54)
ammonium salts as additives, 70 °C	56	(54)
presence of fluorinated additives, 30 °C	67	(29)
presence of oxidizers like SeO_2_, 85 °C	70	(25)
hexafluoroisopropan-2-ol solvent as a radical-stabilizing agent, low temperatures	60	(27)

The film morphology and properties may influence the electrosynthesis of **TMBh** in different ways. The electrosynthesis proceeds in four general steps, including (i) diffusion of substrates to **MIP** cavities, (ii) electron transfer from substrates to the electrode during electro-oxidation, (iii) coupling of the radicals formed, and (iv) diffusion of products out of the cavities. The diffusion steps depend on the film thickness, morphology, and porosity. Electron transfer efficiency depends on the cavity distance from the surface of the electrode, as well as the film conductivity. Clearly, the morphology and thickness of the **MIP-a** and **MIP-b** films are different, affecting the synthesis outcome. Because of this difference, the increased time during which substrates are present within the thicker film may lead to the increased possibility of the side or following reactions, thus, lowering the selectivity of thicker **MIP** films.

Comparison of syntheses performed at various potentials at a **MIP-a** film-coated electrode (Figure 3c) allows concluding that the electrosynthesis performed at 2.00 V vs Ag quasi-reference electrode leads to surprisingly lower **DMPh** substrate conversion and somewhat higher **TMBh** product yield than it is observed at 1.20 V vs Ag quasi-reference electrode. However, prolonged electrosynthesis (longer than 2 h) at the higher potential of 2.00 V vs Ag quasi-reference electrode irreversibly affected the film stability.

On the other hand, the **MIP-a** films exhibited stability in recurring electrosyntheses for up to 2 h (Figure 3d). The **MIP-a** films maintained the conversion and yield for two cycles of 2-h synthesis at 1.20 V vs Ag quasi-reference electrode and for two cycles of 30 min synthesis at 2.00 V. This observation indicates that these **MIP** systems can be applied for large-scale electrosynthesis. Interestingly, the desired **TMBh** product yield was equivalently lower if the **DMPh** substrate concentration was lower (Figure 3e), indicating that decreasing the substrate concentration does not positively affect the desired product yield. Moreover, electrosynthesis on the noncoated Pt electrode did not yield the **TMBh** even at lower **DMPh** substrate concentrations, though the substrate conversion was still high. Most likely, the phenolic **DMPh** substrate degrades or polymerizes at the bare electrodes. This supposition was confirmed by HPLC results (Figure S13 in the Supporting Information) at 280 nm, which do not show significant amounts of products of electro-oxidation at the bare electrode.

The above results indicate that the thinner **MIP-b** film exhibits a slightly higher selectivity toward the preferred **TMBh** product. Furthermore, increasing the electro-oxidation potential is beneficial for obtaining higher selectivity. However, applying a potential exceeding 2 V may lead to film integrity issues and solvent oxidation.

Although the presented approach offers excellent flexibility and facility of electrode preparation together with a more “green” methodology avoiding large amounts of toxic chemicals, it suffers from certain limitations. Since MIP films used in our study are all-organic materials, low resistance to elevated temperatures or highly aggressive chemicals, including concentrated acids or bases, would be their apparent limitations. However, the proposed electrosynthetic approach offers reasonable selectivity and yield even without such extreme conditions. Another possible limitation stems from **MIP** as a heterogeneous catalyst. That could lead to issues with the mass transfer limitations. However, this limitation can be lifted by appropriate reaction conditions design (e.g., using a flow system). The other possible issue is the need to synthesize a dedicated FM required to deposit **MIP** films. This limitation is mitigated by a relatively small FM amount needed to coat large electrodes as the required **MIP** film is relatively thin (≤1 μm). Further optimization may allow the use of cheaper, commercially available monomers capable of electropolymerization. Therefore, our synthetic approach seems advantageous and attractive for industrial applications.

#### 3.4.1 Mass Spectrometry (MS) and UV–vis Spectroscopy Analysis of the Synthesis Product

As mentioned above, the biphenol synthesis typically leads to the formation of various products as highly reactive radicals are formed.^27^ Besides the desired 2–2′ C–C coupling product, other regioisomers can be formed, including 2–3′ or 3–3′ C–C coupling products. Furthermore, dimers, trimers, or even oligomers resulting from the sequential coupling of the **DMPh** monomers can be expected and were observed, indeed. Another expected class of products includes ethers, where the C–O radical is coupling. Further rearrangements can occur following such coupling, leading to the formation of ketones. In the case of the reaction mixture used herein, where a relatively chemically inert solvent and supporting electrolyte are used under anaerobic conditions, various **TMBh** regioisomers, dimers, and trimers, as well as ethers and ketones, are to be expected. After the electrosynthesis, the fractions of the products at 6.7, 11, 14.9, and 19.7 min were separated by HPLC and collected, as well as samples of the **DMPh** and **TMBh** standards were studied by MS to clarify the issue. Furthermore, UV–vis spectra for those compounds were recorded during HPLC separation using a diode array detector.

In all MS spectra obtained, signals from the ClO_4_^–^ ion fragments at *m*/*z* values of 98.94 and 100.94 Da are present (Figure S14 in the Supporting Information). Evidently, the procedure used to remove the supporting electrolyte from the collected fractions was not fully efficient. Nevertheless, the amount of ClO_4_^–^ was low enough to enable the recording of the signals from other negative ions.

The **DMPh** substrate (Figure S14a in the Supporting Information) and the desired **TMBh** product (Figure S14b in the Supporting Information) show dominant peaks at 121.07 and 241.12 Da, respectively, corresponding to **DMPh** and **TMBh** anions with the −OH group proton being removed. This result agrees with a typical fragmentation pattern for phenols.^55,56^ Moreover, MS spectra for both compounds show the ion presence at *m*/*z* of ∼220 Da. Furthermore, ions at *m*/*z* of 322.68 Da for **DMPh** and 283.25 Da for **TMBh** were found. These ions are most likely generated by the phenol moiety further fragmenting and coupling upon ionization.

In the fraction collected at 6.7 min (Figure S14c in the Supporting Information), an intense signal at *m*/*z* = 168.99 Da and three signals at 220.89, 283.26, and 384.97 Da of lower intensity are seen. Hence, the examined molecular mass of this product is lower than that of **TMBh**, closer to that of **DMPh**. A shorter retention time also indicates the formation of a more polar compound, presumably a **DMPh** derivative. At bare electrodes, that was a major product of electrosynthesis.

The MS spectrum of the HPLC fraction collected at 11.0 min (Figure S14d in the Supporting Information) showed dominant *m*/*z* of 283.26 and 241.12 Da signals, accompanied by weaker signals at 144.96 and 168.99 Da and also several less intense signals at 196.98, 220.88, and 255.23 Da. Moreover, it contained weak signals at *m*/*z* exceeding 350 Da (mainly at 410.36 and 537.51 Da), which were supposed products of the primary ions’ coupling. The shorter retention time and a relatively complex fragmentation pattern indicate that this product is a **TMBh** derivative, which contains more polar groups including hydroxyl and carbonyl. Interestingly, it looks as if this compound contained a fragment like that being dominant in the fraction collected at 6.7 min. Also, this product was formed in quite substantial amounts at the bare Pt electrode.

In its MS fragmentation pattern, the HPLC fraction at 14.9 min (Figure S14e in the Supporting Information) is somewhat like the **TMBh** fragmentation spectrum (Figure S14b in the Supporting Information), where the most abundant ion at *m*/*z* of 241.12 Da is very similar to the **TMBh** structure with removed proton. A less abundant ion at a *m*/*z* of 144.97 Da is also observed here. However, the spectrum of the fraction collected at 14.9 min does not contain a signal at *m*/*z* of 220.81 Da, present in the **TMBh** spectrum. In both samples, a low-intensity peak seen at *m*/*z* > 250 Da can arise from products of the primary fragmentation ions coupling. Presumably, both the 14.9 and 14.1 min (**TMBh**) fractions contained structurally similar molecules, probably an isomer of **TMBh**. This inference is supported by their retention times, like that of **TMBh**, being 14.1 min.

The HPLC fraction at 19.7 min (Figure S14f in the Supporting Information) shows a weak MS signal at 253.23 Da and a much more intense at *m*/*z* of 144.97 Da. Moreover, the spectrum reveals several similarly intense peaks at 168.99, 220.89, 241.12, 283.26, and 289.11 Da. The signal at *m*/*z* of 289.11 Da, nearly double the signal at 144.97 Da, indicates a dimer. Furthermore, there is a range of medium-size signals at 504.51, 537.51, and 666.05 Da (not shown), possibly corresponding to primary ions’ coupling products. The fragmentation observed for this fraction, which is more complex than that of **TMBh** and the fraction at 14.9 min, indicates cleaving a more complex molecule, including a moiety like **TMBh**. This MS pattern and the observed longer retention time indicate a higher molar mass and lower polarity of the compound present in this fraction.

The UV–vis spectra for each studied HPLC fraction provided additional information on the nature of the side products (Figure S15 in the Supporting Information). A spectrum of the fraction collected at 6.7 min (Figure S15c in the Supporting Information) shows two overlapping bands at ∼249 and ∼262 nm. Those bands are blue-shifted against a broad band at ∼281 nm for **DMPh** (Figure S15a in the Supporting Information) or bands at 290 and 330 nm for **TMBh** (Figure S15b in the Supporting Information). This shift indicates that the compound contains moieties less conjugated than both the substrate and the product and, presumably, is nonaromatic. The UV–vis spectrum for the fraction collected at 11.0 min (Figure S15d in the Supporting Information) contains a strong band at 203 nm, overlapping with that at 251 nm and two bands at 290 and 328 nm. This spectral pattern resembles that for **TMBh** (Figure S15b in the Supporting Information), where bands at 206, 250, 289, and 330 nm are seen. Moreover, some band position and intensity differences indicate the presence of similar chromophore moieties in both compounds. This result agrees with MS results (Figures S14b and S14d in the Supporting Information), indicating the presence of the **TMBh** moiety in both compounds.

The spectral pattern for the fraction collected at 14.9 min (Figure S15e in the Supporting Information) is even more like that for **TMBh**. The UV–vis spectrum for this compound shows bands at 203, 249, 290, and 330 nm, although the absorbance ratio of the band at 290 to that at 330 nm is higher than that for **TMBh**. The MS results (Figures S14e and S14b in the Supporting Information) show that in both the **TMBh**-containing fraction and the fraction at 14.9 min, the dominant compound ion is deprotonated **TMBh**. Furthermore, both fractions have relatively short retention times. Therefore, one can conclude that the compound collected at 14.9 min is presumably an isomer of the desired **TMBh** product. Finally, the UV–vis spectrum for the fraction collected at 19.6 min (Figure S15f in the Supporting Information) is much more complex, showing at least eight bands at ca. 200, 226, 261, 273, 281, 306, 319, and 343 nm. Those bands are characteristic of electronic transitions in both nonaromatic and aromatic moieties. This result agrees with the complex MS fragmentation pattern of this compound (Figure S14f in the Supporting Information) and supports the inference that this byproduct is quite complex, containing **TMBh** moiety, as well as several other aromatic and nonaromatic moieties.

### 3.5 Theoretical Studies of the Selective **TMBh** Electrochemical Synthesis at Molecularly Imprinted Polymer (**MIP**) Films

**Scheme 3:**
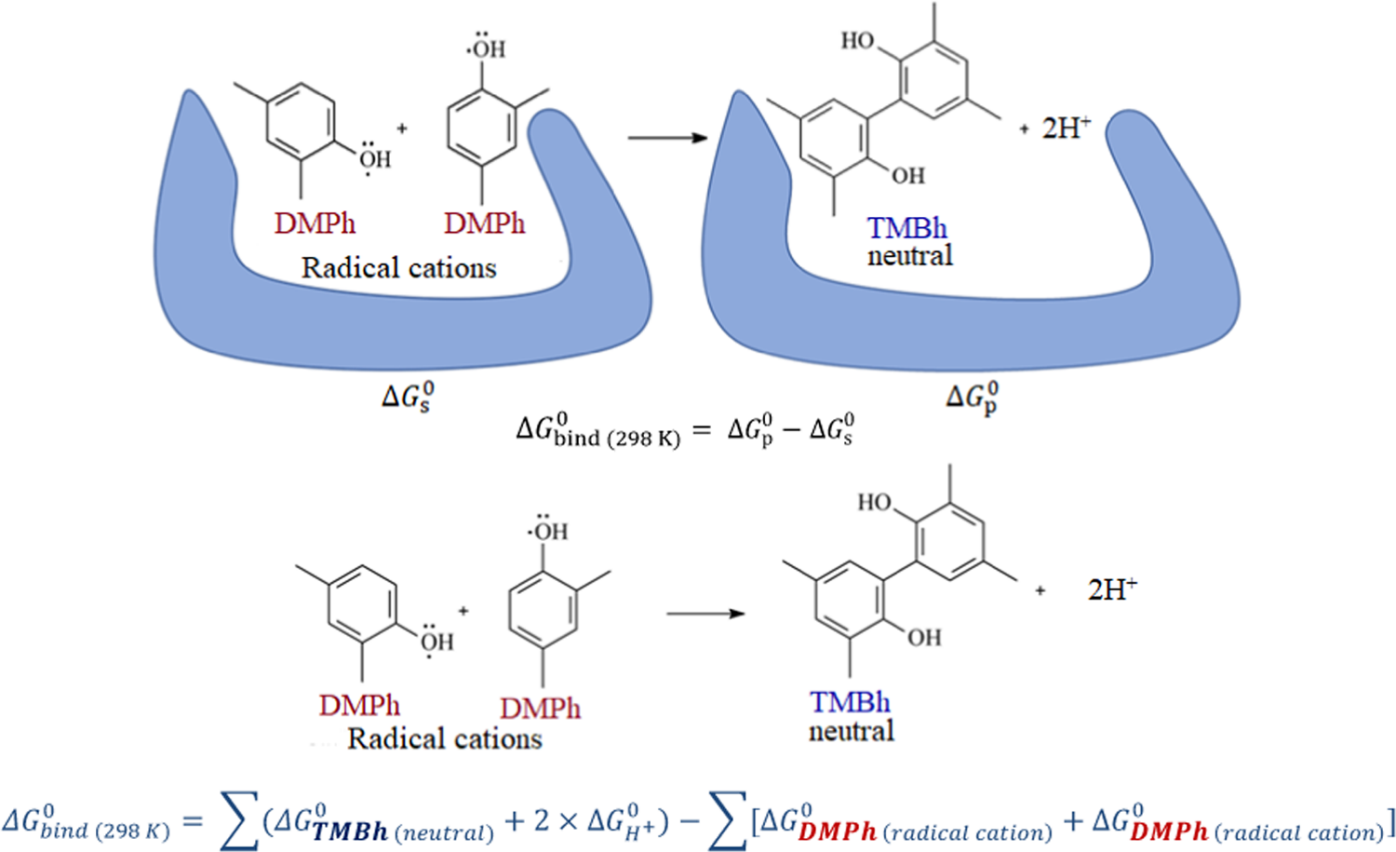
Proposed Mechanism of **DMPh** Electro-Oxidation to the C–C Coupled **TMBh** in the **MIP** Molecularly Imprinted Cavity and Outside of the Cavity The equation shows how these reactions’ standard Gibbs free energy change was calculated.

During the **TMBh** electrosynthesis on the **MIP** film-coated electrodes, the **DMPh** substrate is expected to couple in the **MIP** cavities selectively, i.e., two **DMPh** molecules form radical cations followed by coupling to create one **TMBh** molecule. Computational simulations were applied to elucidate the **MIP**s’ selectivity to this electrosynthesis and explain the process more deeply (Scheme 3).

#### 3.5.1 Insight into the **MIP** Cavity Formation

The **MIP** cavity model was constructed by simulating the electropolymerization of the **BTMA** and the **CM** in the presence of the **TMBh** template (Figure S16 in the Supporting Information). All components’ molecular electrostatic potential (MEP) distribution has been calculated and used to color the cavity surfaces. The positive MEP was formed on the cavity edges, while the neutral MEP was located near the **BTMA** and **CM** thiophene rings. Only slightly negative MEP was positioned close to the benzene rings of **BTMA** and **CM**. Moreover, negative potential sites were placed near the oxygen atoms of the **BTMA** carboxyl group.

#### 3.5.2 Reactions in the **MIP** Cavity

**Figure 4:**
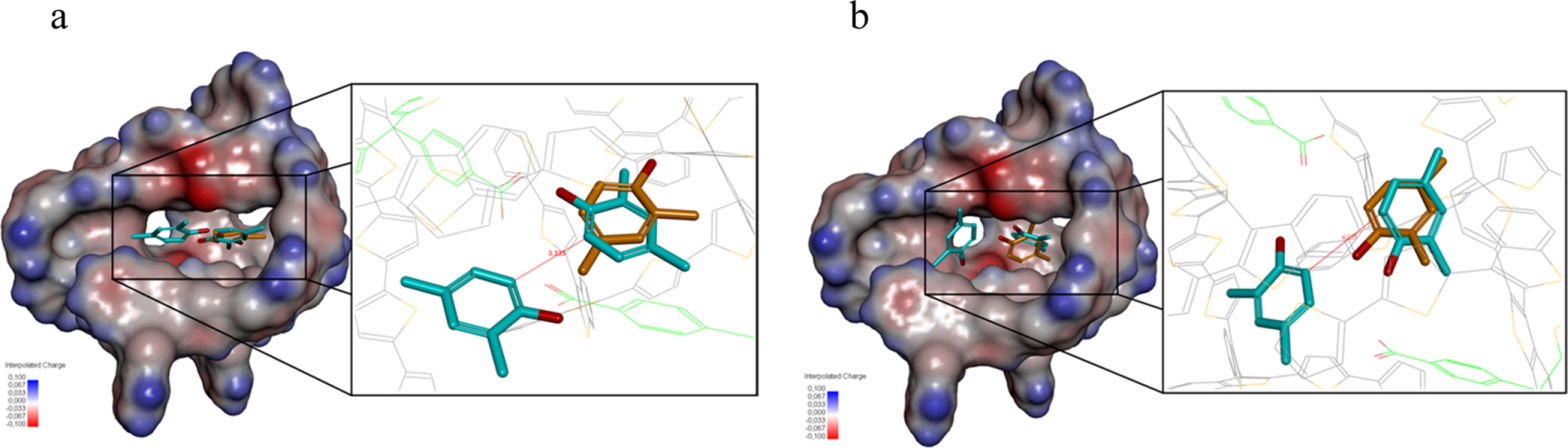
MD simulated substrate molecules’ positions in the **MIP** cavity. (a) One neutral substrate molecule (orange) position in the **MIP** system, as well as the two neutral substrate molecules’ (turquoise) position. (b) One **DMPh**^•+^ molecule (orange) position within the cavity, as well as the two **DMPh**^•+^ molecules’ (turquoise) position.

In order to couple and form **TMBh** during electrosynthesis, the substrate **DMPh** should diffuse from the solution into the cavity. To simulate this process, the **DMPh** molecules were inserted into the cavity one after the other. That allowed for determining the **DMPh** molecules’ arrangement in the cavity and testing if their positions permit **DMPh** conversion into **TMBh** (Figure 4).

The first **DMPh** molecule arranges itself deep in the cavity. Its hydroxyl and *ortho* methyl groups are oriented toward the cavity center, and the *para* methyl group points toward the cavity wall. The second molecule fills the space left in the cavity with its methyl and hydroxyl groups pointing outside it. Noteworthy, entering the second molecule leads to the first molecule orientation change.

The first molecule is still hidden deep in the cavity, albeit its two methyl groups now point to the center of the cavity, and its hydroxyl group is rearranged to face the cavity wall. The distance between C–C atoms for neutral **DMPh** is calculated as 3.33 Å (Figure 4a). The mechanism then involves electro-oxidation of the sorbed neutral **DMPh** molecules to the **DMPh**^•+^ radical cations and then their coupling to the expected **TMBh** product. This process is most likely associated with a few steps of proton removal. The calculations of the first and final steps of the process and their analyses assumed that the intermediate radical cations, the **TMBh** product, and 2H^+^ ions are located inside the cavity one after the other.

Two radical cations of the **DMPh**^•+^ substrate (Figure 4b) occupy the same cavity section as the neutral **DMPh** molecules, although their organization is somewhat different. The first **DMPh**^•+^ locates itself deep in the cavity, with its methyl substituents pointing to the cavity center and the hydroxyl group oriented outside the cavity. The second **DMPh**^•+^ occupies the space left in the cavity, but its methyl moieties point outside of the cavity, and the hydroxyl group is now facing the inside of the cavity. That means that the organization of two substrate molecules inside the cavity can indeed facilitate the C–C bond formation. The distance between carbon atoms of the **DMPh**^•+^ molecules is predicted as 4.03 Å (Figure 4b). The MD calculated Δ*G*_bind_^0^ value is −152.9 kJ mol^–1^ for the neutral substrates in the **MIP** cavity, while it is negatively increased to −183.7 kJ mol^–1^ for the electro-oxidized substrates. That means the system stability increases after substrate electro-oxidation, thus favoring further reaction steps.

#### 3.5.3 QM/MM Calculations of the **MIP** Impact on the Reaction

To elucidate the effect of the cavity presence on the electrosynthesis of **TMBh**, the standard Gibbs free energy changes, Δ*G*_reaction(298K)_^0^ were calculated for the synthesis inside and outside the **MIP** cavity (in a free environment). The calculations were performed by using the QM/MM theory on the DFT/Amber level (Table 2). In the case of the **DMPh** dimerization outside the cavity in the simulated ACN/DCM (9:1, v/v) mixed solvent solution, the Δ*G*_reaction(298K)_^0^ value is highly positive. That indicates that the possibility of dimerization is very low in that case. On the other hand, if the synthesis is performed inside a simulated **MIP** cavity (i.e., on the electrode coated with the **MIP** film), the Δ*G*_reaction(298K)_^0^ value is highly negative. That corroborates the beneficial effect of imprinted cavities on **TMBh** electrosynthesis.

**Table 2:** Standard Gibbs Free Energy and Standard Enthalpy Changes Accompanying the **TMBh** Synthesis

reaction	Δ*G*_reaction(298K)_^0^ (kJ mol^–1^)	Δ*H*_reaction (298 K)_^0^ (kJ mol^–1^)
Free Molecules		
DMPh^•+^ + DMPh^•+^ → TMBh + 2H^+^	+515.5	+519.6
Molecules Bound Inside the MIP Cavity		
DMPh^•+^ + DMPh^•+^ → TMBh + 2H^+^	–573.5	–588.1

#### 3.5.4 Interactions of the **DMPh** Substrate with the **MIP** Cavity

The **MIP** impact on the electrosynthesis pathway can be elucidated by analyzing the interactions between the cavity and the substrate molecules. In Figure S17 in the Supporting Information, the cavity-substrate interactions are presented as dashed segments. The **DMPh**^•+^ forms three hydrogen bonds and two π-donor bonds between the hydroxyl groups of the radical cation and thiophene ring sulfur atoms of one **BTMA** molecule and benzothiophene rings of two **CM** molecules with lengths of 3.54–3.77 Å. The cavity interactions with hydroxyl groups can aid in orienting substrate molecules in a fashion to arrange them to resemble the desired **TMBh** product. Furthermore, the **BTMA** carboxyl groups’ positions in the cavity allow for generating electrostatic interactions of the π-anion type with the aromatic rings of both **DMPh**^•+^ cations (lengths of 4.56 and 4.66 Å), thus supporting the advantageous arrangements of **DMPh**^•+^. Moreover, hydrophobic interactions (π–π sigma, π–π T-shaped, and π-alkyl types) between the thiophene and benzothiophene rings of **CM** and the methyl groups of **DMPh**^•+^ can be observed with lengths of 3.57–5.48 Å. Those interactions further support a favorable molecular orientation.

Hence, the interaction of the substrate molecule with the **MIP** cavity favors the selective electrochemical synthesis of the desired product. This interaction helps to preserve the selectivity of the **MIP** polymer toward **TMBh**.

## 4 Conclusions

The preferred product of electro-oxidation of the 2,4-dimethylphenol **DMPh**, namely, 3,3′,5,5′-tetramethyl-2,2′-biphenol **TMBh**, was first successfully used as the template in the molecularly imprinted polymer (MIP). The MIP preparation exploited the electrostatic and hydrogen bond interactions between the **TMBh** template and the functional monomer, **FM**, containing a carboxyl group. The *p*-bis(2,2′;5′,2″-terthien-5′-yl)methylbenzoic acid **BTMA** was selected as the most efficient **FM** among those examined based on the performed DFT calculations. The **FM** deprotonation appeared beneficial for the complex formation because of the strong bond between the carboxylate of **BTMA** and **TMBh** at the 1:2 molar ratio of **TMBh**: **FM**. The FTIR and UV–vis spectroscopy measurements confirmed interactions between prepolymerization complex components.

Subsequently, the **MIP** film was fabricated on the electrode by a potentiodynamic electropolymerization. The **MIP** and **NIP** films were rough and relatively thick (400–900 nm) with softer and more rigid domains, as attested by nanomechanical properties mapping. Then, the **TMBh** acting as a template was successfully removed from the **MIP** cavities, as confirmed by PM-IRRAS experiments performed before and after template extraction, as well as by using the “gate effect” with DPV measurements of the ferrocene redox probe signal.

In the final step, the electrodes coated with the **MIP** film were employed for the **TMBh** electrosynthesis. The selectivity of reaction toward the preferred C–C coupled product performed at the **MIP** film-coated electrodes was higher than at the bare electrodes or electrodes coated with **NIP** film. When the thinner **MIP-b** film was used, the selectivity of the electrosynthesis at 1.20 V vs Ag quasi-reference electrode reached 39%. This polymer was appreciably stable, as it preserved the yield and selectivity during the second electrosynthesis. When a higher electrode potential was applied, the reaction yield increased, although the poisoning of the film by the products at a longer (exceeding 2 h) electrosynthesis time became evident. Therefore, there is a trade-off between the high selectivity of the reaction and the stability of the **MIP** film.

Computational studies showed that the Gibbs free energy change of the coupling reaction in the cavity was negative compared to the Gibbs free energy change of this reaction outside the cavity, explaining the selectivity of the **MIP**. The analysis of the calculated structures confirmed that the **MIP** cavity helped to orient two substrate molecules, promoting C–C coupling and leading to the formation of **TMBh**.

Combining electrochemical synthesis with the **MIP** technology allowed for a significant increase in the selectivity of the phenol C–C coupling, leading to the desired biphenol product without the need to increase the temperature of the process or use harmful additives.

## Supporting Information Available

List of used compounds, scheme and procedure of the *p*-bis(2,2′;5′,2″-terthien-5′-yl) methylbenzoic acid (**BTMA**), detailed description of the instruments used, custom-made three-electrode V-shaped electrochemical glass cell cross-sectional view, standard Gibbs free energy change (Δ*G*^0^) accompanying prepolymerization complexes formation, current–potential curves of diphenylamine-2-carboxylic acid potentiodynamic electrochemical polymerization, experimental and simulated UV–vis spectra for **TMBh** complexation with **BTMA**, FTIR spectroscopic analysis of **TMBh** complexation with **BTMA**, PM-IRRAS and GA-FTIR spectra for Au electrodes coated with **MIP-b** and **NIP-b** film before treatment with the extracting solution and after it, AFM images of the thick **MIP-a** films, AFM images of the thin **MIP-b** films, results of AFM analysis of **MIP** films before **TMBh** extraction and after it and **NIP** films before and after immersion in the extracting solution, **MIP** and **NIP** films maps of Young’s modulus, CV of 2,4-dimethylphenol, CV curves recorded during pretreatment of the **MIP-a** film deposited on Pt electrode, charge vs electrosynthesis time curves recorded during electro-oxidation of **DMPh**, HPLC chromatograms for the **DMPh** substrate and the desired **TMBh** product, exemplary HPLC chromatograms for fractions collected after 14 h electrosynthesis, mass spectra of the reaction mixture fractions and **DMPh** and **TMBh**, reaction mixture fractions UV–vis spectra and spectra of **DMPh** and **TMBh**, computationally simulated structure of the molecular cavity of MIP, and computationally simulated interactions of two **DMPh**^**•+**^ substrate radical cations in the simulated MIP cavity model (PDF)
