# Supplementary material for: Improving the Selectivity of the C–C Coupled Product Electrosynthesis by Using Molecularly Imprinted Polymer—An Enhanced Route from Phenol to Biphenol
Source: ACS Appl Mater Interfaces. 2023 Oct 12;15(42):49595–610. doi: 10.1021/acsami.3c09696 (PMC10614056; doi:10.1021/acsami.3c09696)
Supplement: Supplementary file 1 — am3c09696_si_001.pdf [file am3c09696_si_001.pdf]

Supporting information

Improving the Selectivity of the C-C Coupled Product Electrosynthesis by Using Molecularly Imprinted Polymer - An Enhanced Route from Phenol to Biphenol

Alcina Johnson Sudagar,[a] Shuai Shao,[b] Teresa Żołek,[c] Dorota Maciejewska,[c] Monika Asztemborska,[a] Maciej Cieplak,[a] Piyush Sindhu Sharma,[a] Francis D'Souza,[b] Włodzimierz Kutner,[a,d] and Krzysztof R. Noworyta*,[a]

[a] Institute of Physical Chemistry, Polish Academy of Sciences, Kasprzaka 44/52, 01-224 Warsaw, Poland.

[b] Department of Chemistry, University of North Texas, Denton, 1155, Union Circle, #305070, TX 76203-5017, U.S.A.

[c] Department of Organic and Physical Chemistry, Faculty of Pharmacy, Medical University of Warsaw, Banacha 1, 02-097, Warsaw, Poland.

[d] Faculty of Mathematics and Natural Sciences, School of Sciences, Cardinal Stefan Wyszynski University in Warsaw, Wóycickiego 1/3, 01-815 Warsaw, Poland.

*****Correspondence: knoworyta@ichf.edu.pl (K.R.N.)

Table of content

**Table S1**.	List of compounds	S4
**Section S1**	Procedure of synthesis of *p-bis*(2,2';5',2"-terthien-5'-yl) methylbenzoic acid (**BTMA**)	S5
**Scheme S1.**	Scheme of the reaction used for the preparation of *p-bis*(2,2';5',2"-terthien-5'-yl) methylbenzoic acid (**BTMA**)	S6
**Section S2**	Detailed description of the used instruments	S7
**Scheme S2**.	The cross-sectional view of the three-electrode V-shaped electrochemical glass minicell	S11
**Table S2**.	Standard Gibbs free energy gain (ΔG^0_{bind}) accompanying formation of pre-polymerization complexes	S12
**Figure S1**.	Current-potential curves for the potentiodynamic electropolymerization of diphenylamine-2-carboxylic acid, **DACA**	S14
**Figure S2**.	Experimental and simulated UV-vis spectra for TMBh complexation with FM at the **TMBh**	S14
**Figure S3**.	FTIR spectroscopic analysis of **TMBh** complexation with FM	S15
**Figure S4**.	FTIR spectral region of interest for **TMBh** complexation with FM	S16
**Figure S5.**	PM-IRRAS and GA-FTIR spectra for **MIP-b** and **NIP-b** film-coated Au electrodes before and after extraction	S17
**Figure S6.**	Atomic force microscopy images of the thick **MIP-a** films	S18
**Figure S7.**	Atomic force microscopy images of the thin **MIP-b** films	S19
**Table S3**.	AFM analysis of morphological and nanomechanical parameters of MIP and NIP films before and after **TMBh** extraction	S20
**Figure S8**.	Young modulus maps of MIP and NIP films	S21
**Figure S9.**	The cyclic voltammogram of 2,4-dimethylphenol	S22
**Figure S10**.	CV curves recorded during pre-treatment of the MIP-a film-coated Pt electrode	S23
**Figure S11**.	The charge passed as a function of electrosynthesis time during electro-oxidation of **DMPh**	S23
**Figure S12**.	HPLC chromatograms for the **DMPh** substrate and the desired **TMBh** product.	S24
**Figure S13**.	Exemplary HPLC chromatograms for fractions collected after 14-h electrosynthesis	S25
**Figure S14.**	Mass spectra of fractions of the reaction mixture and **DMPh** and **TMBh**	S26

Figure S15. UV-vis spectra of fractions of the reaction mixture and **DMPh** and **TMBh** S27

Figure S16. A computationally simulated structure of the molecular cavity of MIP S28

Figure S17. The computationally simulated interactions of two **DMPh**$^{\bullet+}$ substrate radical cations in the skeleton model of the MIP cavity S28

Table S1. List of compounds.

Name	Designation	Structural formula	Purpose
2,4-Dimethylphenol	**DMPh**	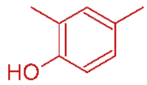	Electro-oxidation substrate
3,3', 5,5'- Tetramethyl-2,2'-biphenol	**TMBh**	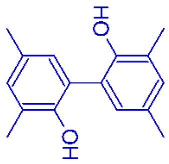	The desired product of electro-oxidation; the template for **MIP**s
Dipehylamine-2-carboxylic acid	**DACA**	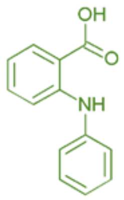	Commercial functional monomer
p-Bis(2,2';5',2''-terthien-5'-yl) methylbenzoic acid	**BTMA**	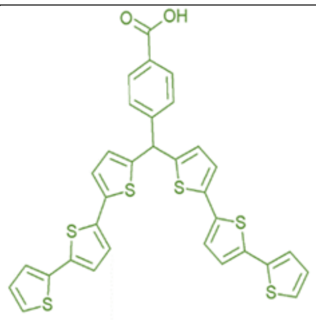	Functional monomer for **MIP** preparation
2,2'-*Bis*(2,2'-bithiophene-5-yl)-3,3'-bithianaphthene	**CM**	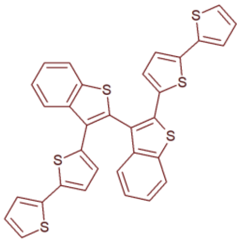	Cross-linking monomer for **MIP**
Triethylamine	TEA	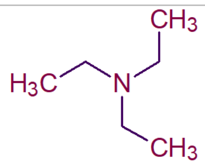	Deprotonating agent for **FMs** in **MIP**s
Tetrabutylammonium perchlorate	(TBA)ClO_4	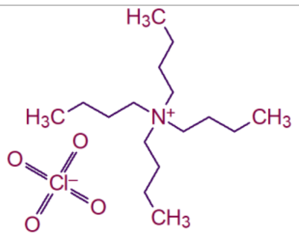	Electrolyte salt
Ferrocene		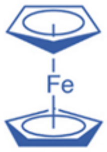	Redox probe

Section S1 *Synthesis of p-bis(2,2';5',2''-terthien-5'-yl) methylbenzoic acid functional monomer (**BTMA**)*

The BTMA synthesis is represented in Scheme S1 in Supporting Information. 2,2':5',2''-Terthiophene (1.54 g, 6.2 mmol), 4-formylbenzoic acid (465.4 mg, 3.1 mmol), and ethylene glycol (50 mL) were stirred in a 100-mL round-bottom flask at room temperature under N_2 atmosphere. After 30 min., 70% $HClO_4$ (6.7 mL) was dropwise into this flask. In effect, the solution color changed from colorless to pinkish-white. The mixture was then stirred at 60 °C under N_2 for 16 h. Afterward, the mixture was dissolved in chloroform and washed with a saturated $NaHCO_3$ solution, then water. The organic layer was dried over anhydrous Na_2SO_4. All the solvents were removed under decreased pressure. The crude product was purified over an LC silica column using hexane, then a series of hexane : chloroform mixtures with increased chloroform content and then pure chloroform as the eluent. The target compound was a light greenish solid. The reaction yield was 1.09 g (55.9%). ^{1}HNMR (400 MHz, $CDCl_3$) δ = 8.04-8.09 (d, J = 8.4 Hz, 2H; Ar-H), 7.43 - 7.48 (d, J = 8.5 Hz, 2H; Ar–H), 7.20 - 7.23 (dd, J = 5.1, 1.1 Hz, 2H; Ar–H), 7.14 - 7.17 (dd, J = 3.6, 1.1 Hz, 2H; Ar–H), 7.04 - 7.06(d, J= 3.8 Hz, 2H; Ar–H), 6.98 - 7.03 (m, 6H; Ar–H), 6.75 - 6.79(d, J = 3.7, 0.7 Hz, 2H; Ar–H), 5.83 (s, 1H; CH–H). ^{13}C NMR (101 MHz, $CDCl_3$): δ = 166.55, 148.04, 145.06, 137.05, 137.02, 136.27, 135.88, 130.19, 129.03, 128.47, 127.87, 127.24, 124.50, 124.27, 124.21, 123.68, 123.12, 66.72, 61.49, 47.69. Absorbance λ_{max}: 240 nm and 369 nm. ATR-FTIR: ~3500 cm^{-1} (OH of carboxyl group stretching), ~3100 cm^{-1} (=CH stretching), ~2900 cm^{-1} (-CH stretching), ~1750 cm^{-1} (C=O stretching), ~1600 cm^{-1} (C=C stretching) ~1440 cm^{-1} (O-H bending), ~900 cm^{-1} (C=C bending), ~800 cm^{-1} (C-H bending).

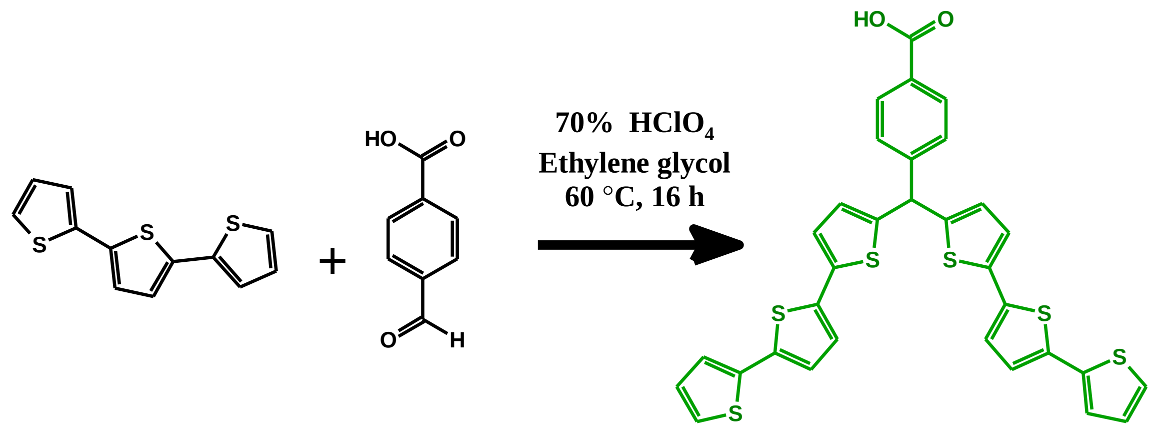

Scheme S1. Synthesis reaction equation for the preparation of *p*-*bis*(2,2';5',2"-terthien-5'-yl) methylbenzoic acid (**BTMA**).

Section S2 *Detailed description of the used instruments*

An expanded and more detailed instrumentation description is given here. A Bio-Logic SAS SP-300 potentiostat/galvanostat electrochemistry system controlled by EC-Lab v.10.37 software of the same manufacturer was used for the potentiodynamic electropolymerization, differential pulse voltammetry (DPV) measurements, polymer conditioning, and potentiostatic electrosynthesis. MIP films and electrosynthesis were preliminarily characterized and optimized using soft-glass shrouded Pt disk working electrodes with a 0.44 mm^2 area. The electrosynthesis was upscaled using Pt plate working electrodes with a 2.74 cm^2 area. The active surface area in electrosynthesis was 1.90 cm^2. A silver wire was used as the quasi-reference electrode, with its potential calibrated using a ferrocene ($C_{10}H_{10}Fe$) internal redox probe. A 4-mm diameter 45-mm long graphite rod was used as the counter electrode. The Shimadzu UV-2501 spectrophotometer was used to record UV-vis spectra of the pre-polymerization complex solution and the solution components with 0.1-nm resolution. All spectra have been recorded using an acetonitrile (ACN) : dichloromethane (DCM) (9 : 1, *v*/*v*) solution. For comparison, spectra of 1 : 1 and 2 : 1 **BTMA** : **TMBh** mixture were calculated from spectra of pure **BTMA** and **TMBh** using Lambert-Beer law and compared to experimental spectra recorded for those mixtures in solution. This procedure allowed finding regions where experimental and calculated spectra changes occurred. The transmission FTIR spectroscopy measurements were performed using a Bruker Vertex 80v spectrophotometer equipped with a DTGS detector to understand the binding within the pre-polymerization complex. The spectra were recorded on ZnSe windows coated with drop-cast samples. The pre-polymerization complex solution and solutions of individual components of MIP were prepared using the acetonitrile (ACN) : dichloromethane (DCM) (9 : 1, *v/v*) solution. The FTIR spectra of the MIP and NIP films before and after exposure to (acetic acid) : methanol extraction solution were recorded with a

Bruker Vertex 80v FTIR spectrophotometer using polarization-modulation infrared reflection-absorption spectroscopy (PM-IRRAS). The spectrophotometer was equipped with a PMA50 module for those experiments. A (liquid nitrogen)-cooled MCT (Hg-Cd-Te) detector was used to reach a reasonably high signal-to-noise ratio. Grazing angle FTIR (GA-FTIR) spectroscopy was used to characterize the MIP-a films deposited on Au-layered glass slides under potentiodynamic conditions. The measurements were performed using the same Bruker Vertex 80v spectrophotometer with GS19650 grazing-angle accessory of SPECAC at the 70° incident angle with pressure decreased to 6 hPa in the sample compartment. FTIR spectra of the synthesized functional monomer were recorded using a single reflection attenuated total reflection (ATR) Platinum accessory of Bruker mounted in the same FTIR spectrophotometer. OPUS 7 software of Bruker was used to analyze all IR spectra.

Nuclear magnetic resonance (NMR) ^{13}C and ^{1}H spectra of functional monomers were recorded using an Agilent DD2 400 MH spectrometer.

Before and after exposure to extraction solution, the MIP and NIP films were also imaged by atomic force microscope (AFM) in the Tapping™ or PFQNM mode with a MultiMode 8 AFM of Bruker controlled by a Nanoscope V controller. A p-doped Si cantilever and tip (RTESP) with a spring force constant of 58.3 N/m were used for measurements. Simultaneously, film topography and nanomechanical properties were measured. Further, the AFM analysis, which included the determination of film roughness, thickness, and phase changes within the film, was performed using NanoScope Analysis v.1.2 software of Bruker. The Derjaguin-Muller-Toporov model was used to calculate the Young modulus and unravel the nanomechanical properties.

The electrosynthesis products were analyzed using an analytical high-performance liquid chromatograph (HPLC) system from Shimadzu Corp. (Kyoto, Japan) with a gradient chromatography set consisting of a DGU-20A degassing unit, LC-20AT liquid

chromatograph to control the gradient mobile-phase solutions and an SPD-M20A UV-vis diode array detector. This system also contained the SIL-20A autosampler of the same manufacturer. The compounds' mixtures were separated using a Luna® 5µm C18(2) 100 Å reversed-phase liquid chromatography column (250 × 4.6 mm i.d., Torrance CA, USA). A mobile phase composed of ultrapure water of 18.2 MΩ cm resistivity at 25 °C and a total organic carbon (TOC) value below 5 ppb (Solvent A) and ACN (Solvent B) was used for gradient elution. A linear gradient was used from a 50 : 50 ratio (Solvent A : Solvent B, *v*/*v*) to a 5 : 95 ratio at 20 min. Reference compounds of the **DMPh** substrate and the desired **TMBh** product were used for identifying compounds and calibrating chromatograms. An FRC-10A fraction collector from Shimadzu attached to the HPLC system was used to collect different fractions of the reaction mixture at retention times of 6.7, 11.0, 14.1, 14.9, and 19.7 min. These fractions were then passed through a silica gel 60 (0.040 – 0.063 mm, mesh 230 – 400, Merck KGaA) LC 50-mm long homemade column with a 10 mm diameter using DCM as the mobile phase to remove the supporting electrolyte from the solution. Afterward, the DCM was evaporated from the eluate, and the solid left was resuspended in 1 mL of an ACN : DCM (9 : 1, *v*/*v*) solution. The reference **DMPh** compound was used directly after diluting with the ACN : DCM (9 : 1, *v*/*v*) solution for the mass spectrometry (MS) analysis.

MS analyses were performed using a Synapt G2-S mass spectrometer (Waters, Milford, MS, USA) equipped with an atmospheric pressure chemical ionization (APCI) system and a quadrupole-time-of-flight (qTOF) mass analyzer. Samples were dissolved in methanol (Honeywell, LC-MS Chromasolv™, purity ≥ 99.9%) and then injected into the APCI ion source. The injection volume was 1 to 8 µL, depending on the concentration of the samples. Methanol with a 100 µL/min flow rate was used as the mobile phase. The measurement was performed in the negative ion mode with the resolving power of the qTOF analyzer at 20,000 full widths at half maximum. The lock-spray source generated the lock-spray spectrum of

Leucine-enkephalin, and the recorded spectra in the range of *m*/*z* = 50 – 1200 were corrected. The desolvation and cone gas used was nitrogen, and their flow rates were set to 600 and 100 L/h, respectively. The ion source and probe temperatures were 120 and 550 °C, respectively. The nebulizer gas pressure was 5.0 bar. The corona current was 12.0 µA, and the sampling cone voltage and source offset were 30 V. The instrument was controlled, and the data were processed using the MassLynx V4.1 software package (Waters).

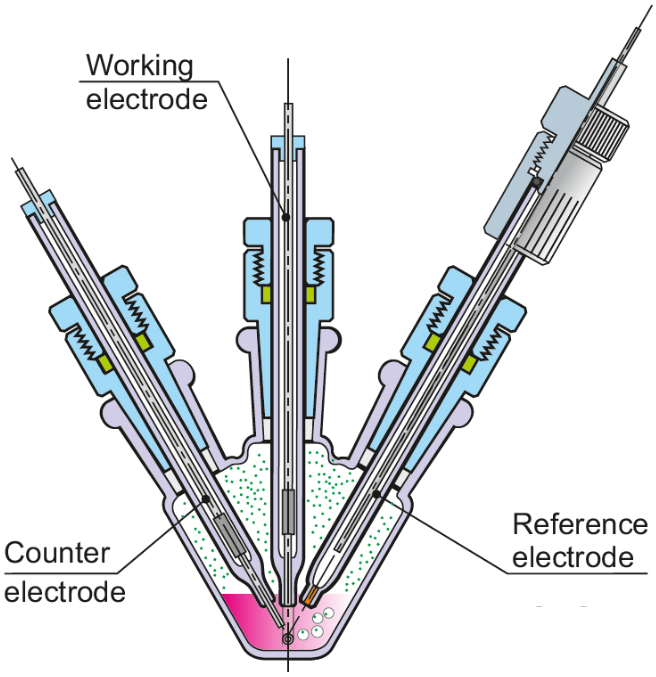

Scheme S2. The cross-sectional view of the three-electrode V-shaped electrochemical glass minicell designed in the Institute of Physical Chemistry, Polish Academy of Sciences (Warsaw, Poland) and custom-manufactured by Labit Sp. z o.o. (Stare Babice, Poland).

Table S2. Standard Gibbs free energy gain (ΔG^0_{bind}) accompanying formation of pre-polymerization complexes of **TMBh** with **FM**, optimized using the DFT B3LYP/6-31g(d) functional and basis set for the complexes in a vacuum.

**Template**<br>**TMBh**	**Functional monomer**<br>**FM**	**Complex stoichiometry**<br>**TMBh : FM**	ΔG^0_{bind}<br>**kJ mol^{-1}**
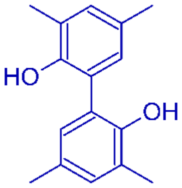<br>**TMBh**	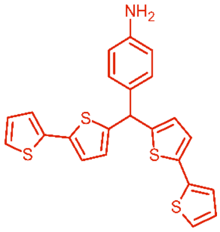<br>*p-Bis*(2,2'-bithien-5-yl)methylaniline	1 : 1	+21
	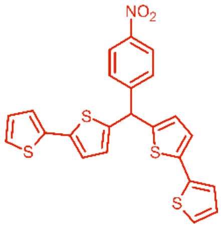<br>*p-Bis*(2,2'-bithien-5-yl)methylnitrobenzene	1 : 1	+15
	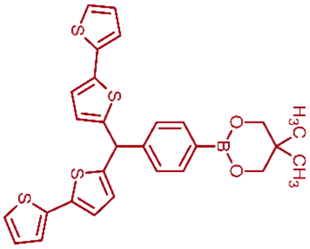<br>*p-Bis*(2,2'-bithien-5-yl)methylphenylboronic acid neopentylglycol ester	1 : 1	+26
	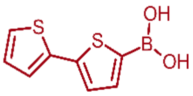<br>2,2'-Bithiophene-5-boronic acid	1 : 1	+2
	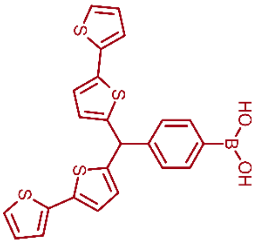<br>*p-Bis*(2,2'-bithien-5-yl)methylphenylboronic acid	1 : 1<br>1 : 2	+1<br>–1

**Template**<br>**TMBh**	**Functional monomer**<br>**FM**	**Complex stoichiometry**<br>**TMBh : FM**	$\Delta G^{0}_{\mathbf{bind}}$<br>**kJ mol^{-1}**
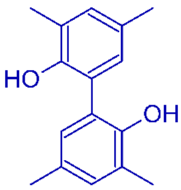<br><br>**TMBh**	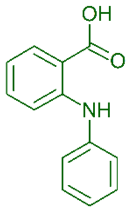<br><br>Diphenylamine-2-carboxylic acid<br>(**DACA**)	1 : 1	+6
		1 : 2	+29
		The carboxylate form interacts with Na^+ for charge neutralization	
		1 : 1	–85
		1 : 2	–182
	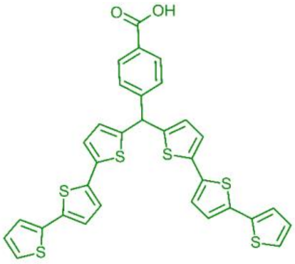<br><br>*p-Bis*(2,2';5',2''-terthien-5'-yl)methylbenzoic acid<br>(**BTMA**)	1 : 1	–9
		1 : 2	–9
		The carboxylate form interacts with Na^+ for charge neutralization	
		1 : 1	–63
		1 : 2	–68

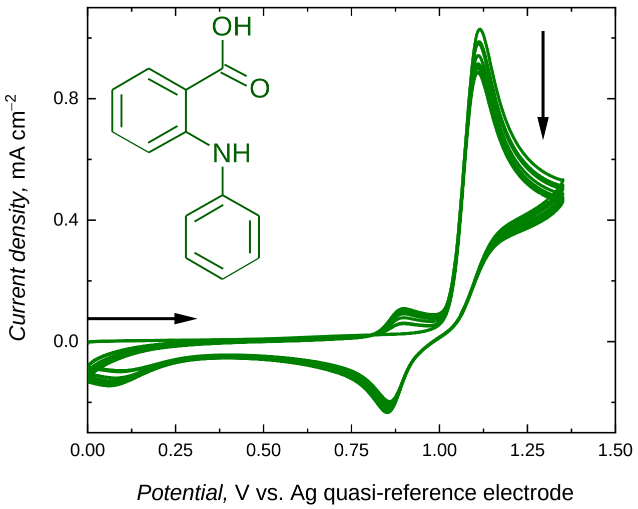

Figure S1. Current-potential curves for the potentiodynamic electropolymerization of 1 mM diphenylamine-2-carboxylic acid, **DACA,** recorded at a Pt disk bare electrode at a scan rate of 100 mV s^{-1} and 100 mM (TBA)ClO_4 in ACN.

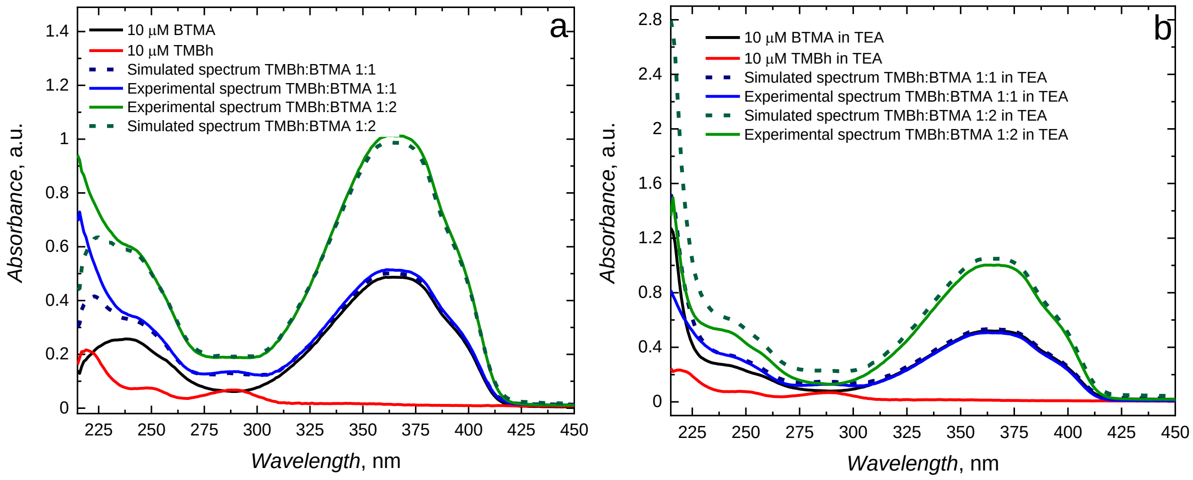

Figure S2. Experimental and simulated UV-vis spectra for **TMBh** complexation with **BTMA** at the **TMBh : BTMA** molar ratios of 1 : 1 and 1 : 2 compared with the spectra for **TMBh** and in the (**a**) absence and (**b**) presence of 1 mM TEA. Spectra were recorded using the acetonitrile (ACN) : dichloromethane (DCM) (9 : 1, *v*/*v*) solution.

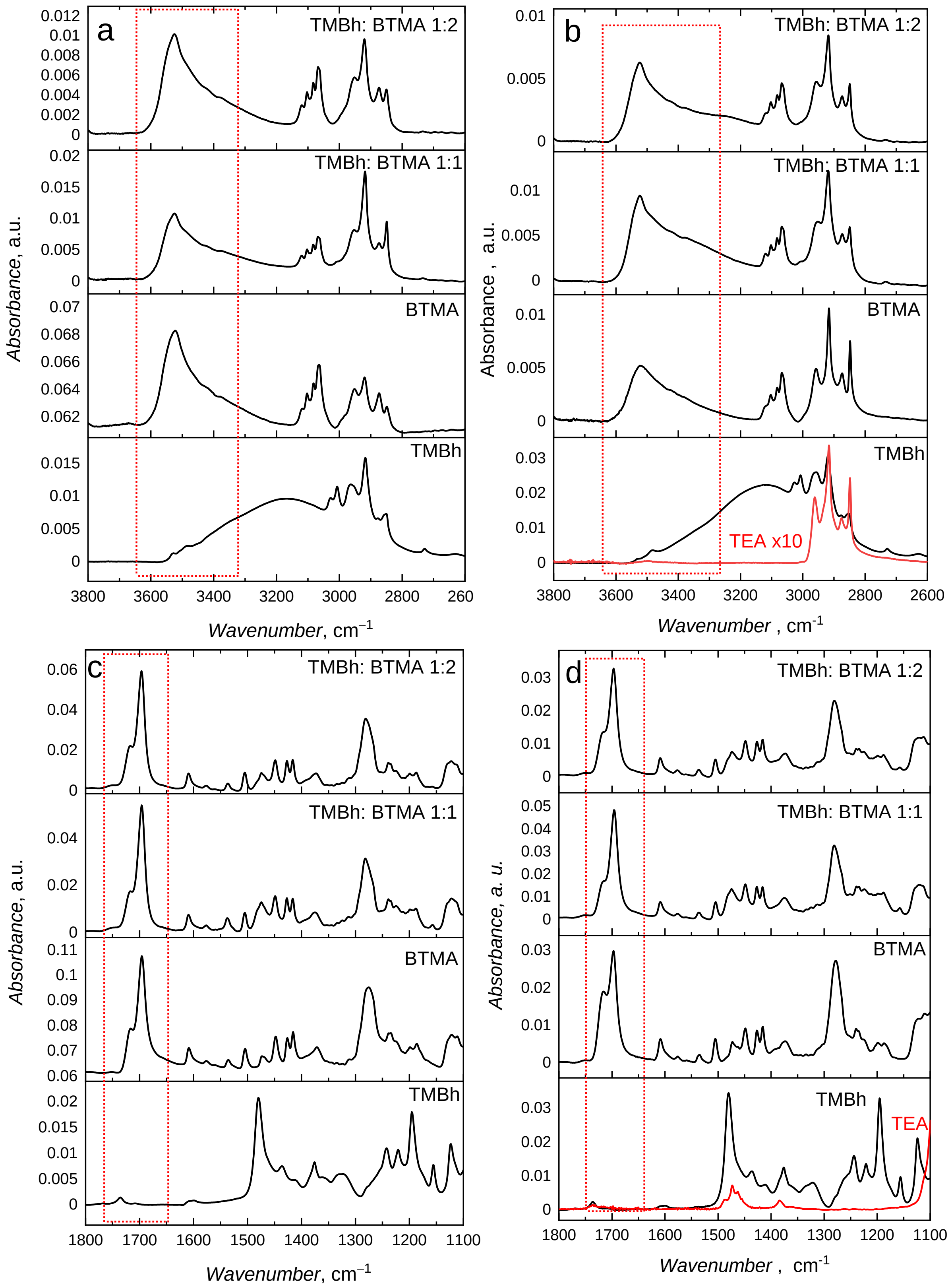

Figure S3. FTIR spectroscopic analysis of **TMBh** complexation with **BTMA** in the (**a**, **c**) absence and (**b**, **d**) presence of 1 mM TEA.

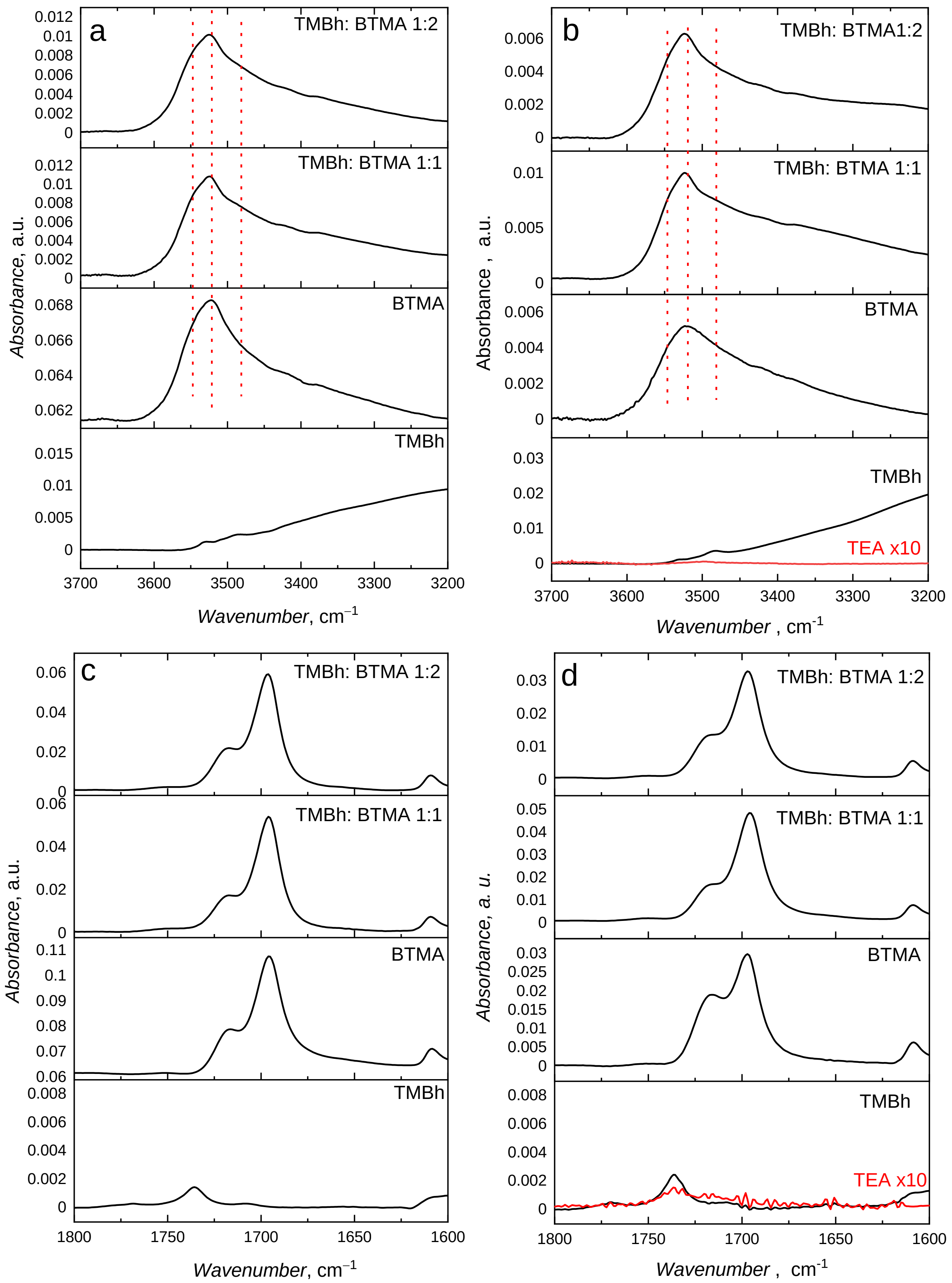

Figure S4. FTIR spectra of **TMBh** complexation with **BTMA** in the (**a**, **c**) absence and (**b**, **d**) presence of 1 mM TEA in selected regions.

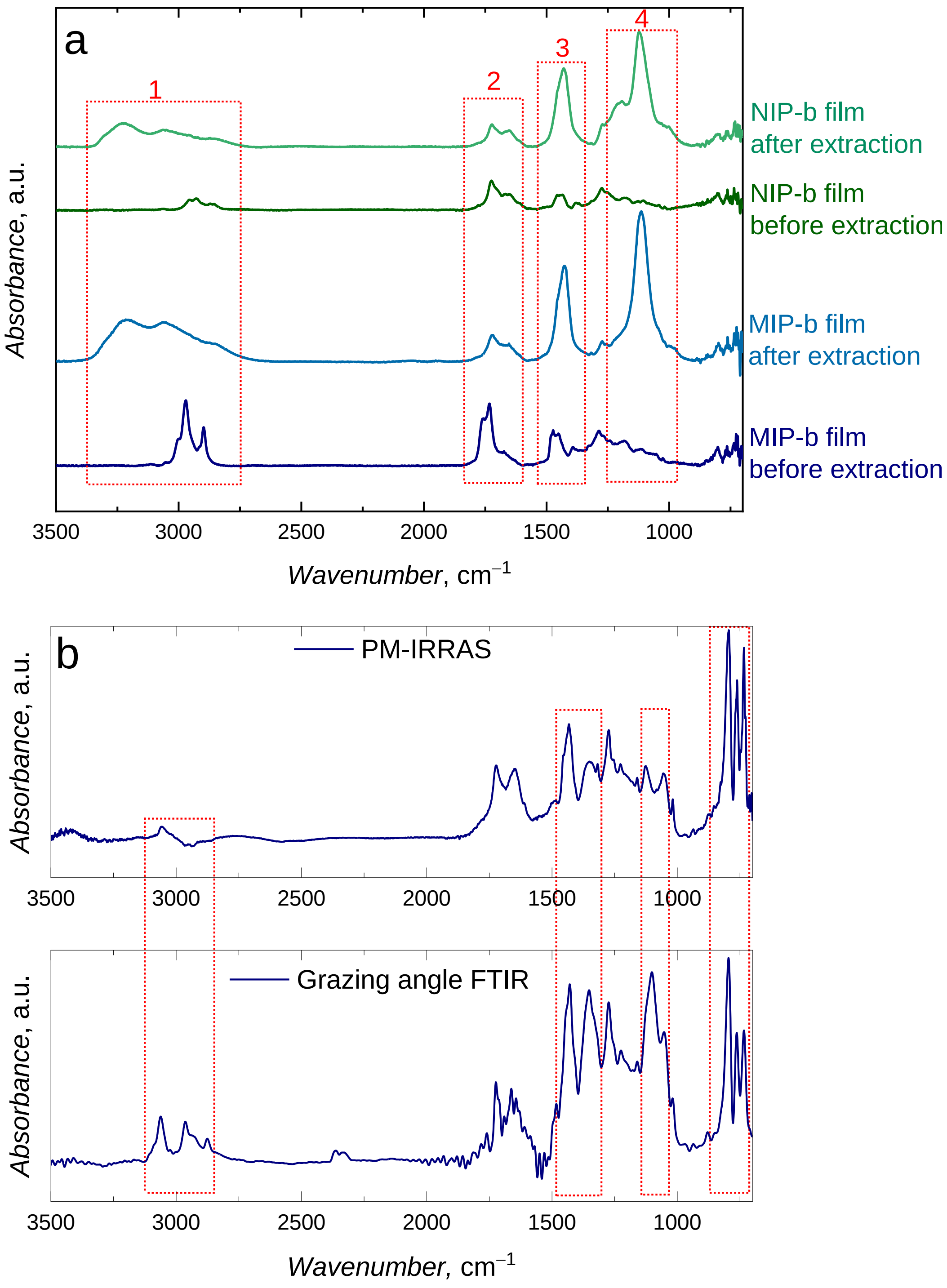

Figure S5. (**a**) PM-IRRAS spectra for **MIP-b** and **NIP-b** film-coated Au electrodes before and after **TMBh** template extraction/incubation for 180 minutes with the (acetic acid): methanol (1 : 1, *v*/*v*) solution and (**b**) comparison of PM-IRRAS and grazing angle FTIR spectra for **MIP-a** film-coated Au electrodes before template extraction.

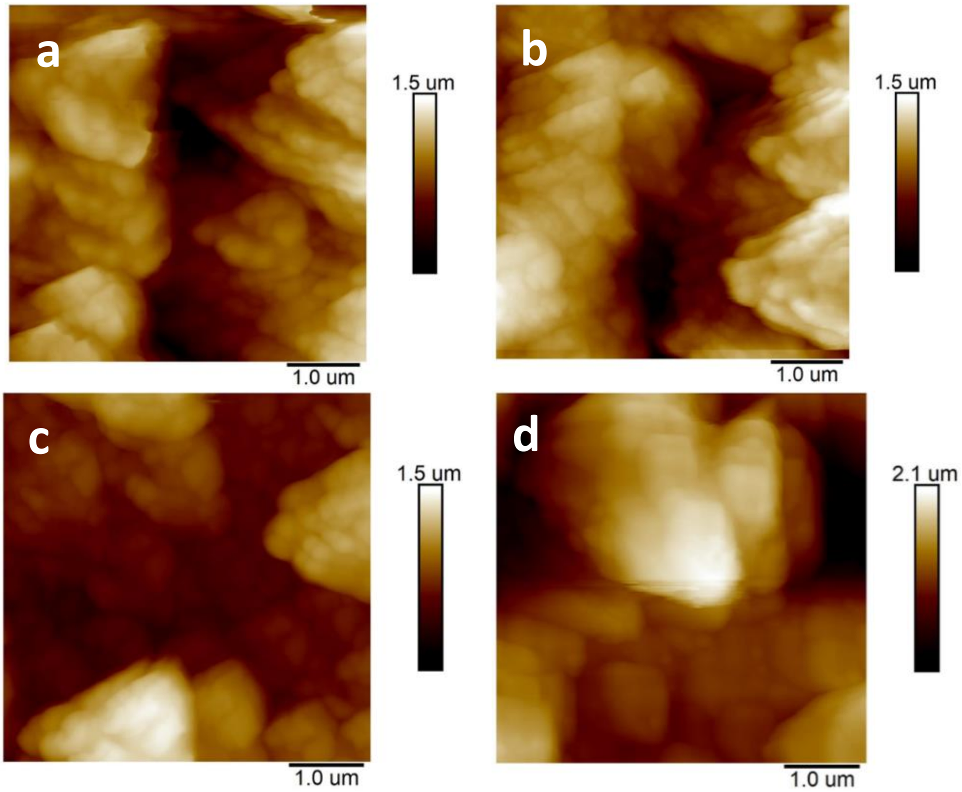

Figure S6. Atomic force microscopy images of the thick **MIP-a** films (**a**) as prepared and (**b**) template-extracted, as well as **NIP-a** films (**c**) as prepared and (**d**) incubated in extraction solution. All films were deposited on Au-layered glass slides using potentiodynamic electropolymerization with five consecutive potential cycles at 50 mV s^{-1} in the acetonitrile : dichloromethane (9 : 1, *v/v*) solution of 200 μM **TMBh**, 400 μM **BTMA**, 1000 μM **CM**, 1000 μM TEA, and 100 mM $(TBA)ClO_4$. The template was extracted in the (acetic acid) : methanol (1 : 1, *v*/*v*) solution for 180 min.

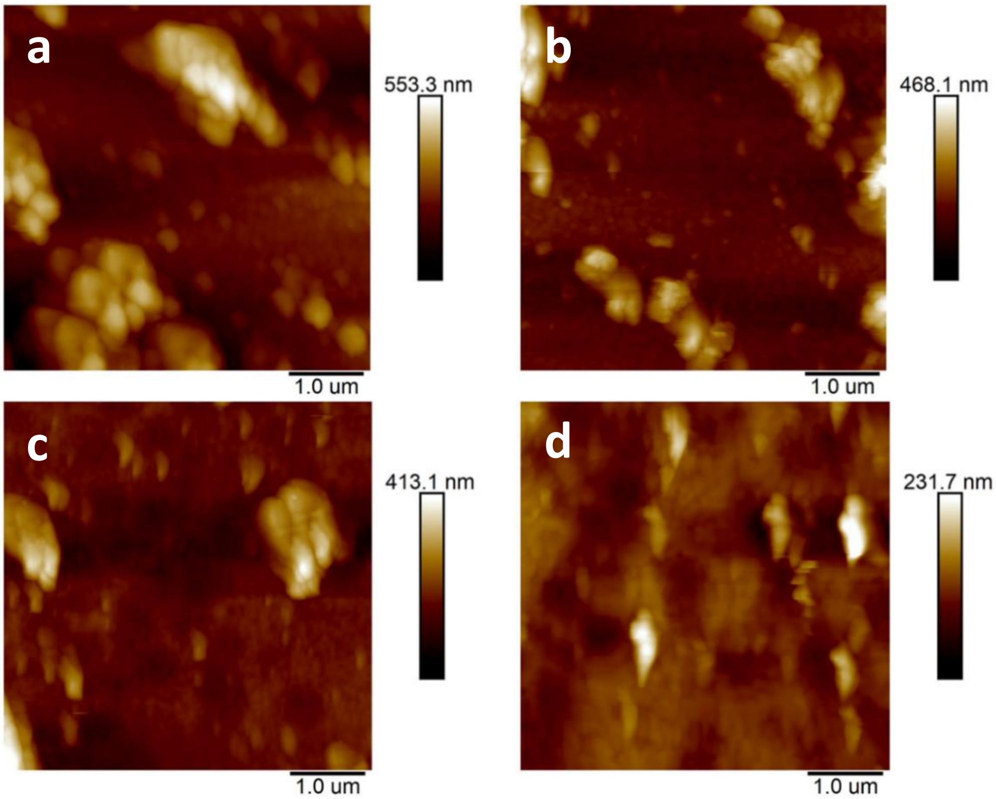

Figure S7. Atomic force microscopy images of the thin **MIP-b** films (**a**) as prepared and (**b**) template-extracted, as well as **NIP-b** films (**c**) as prepared and (**d**) incubated in extraction solution. All films were deposited by potentiodynamic electropolymerization on Au-layered glass slides during five consecutive potential cycles at 50 mV s^{-1} in the acetonitrile : dichloromethane (9 : 1, *v/v*) solution of 20 μM **TMBh**, 40 μM **BTMA**, 100 μM **CM**, 100 μM TEA, and 100 mM (TBA)ClO_4.

Table S3. AFM analysis of morphological and nanomechanical parameters of **MIP** and **NIP** films before and after **TMBh** extraction/incubation for 180 min. in the (acetic acid) : methanol (1 : 1, *v*/*v*) solution.

Polymer film	Extraction condition	Film thickness, nm	Film roughness, R_a, nm	Young modulus, GPa	Dissipation, keV
**MIP-a**	Before extraction	657 ± 54	205 ± 70	0.87 ± 0.42	33.0 ± 10.5
	After extraction	900 ± 104	255 ± 60	1.15 ± 0.39	62.0 ± 38.8
**MIP-b**	Before extraction	488 ± 18	45 ± 14	1.11 ± 0.23	NA
	After extraction	355 ± 21	51 ± 21	1.90 ± 0.09	2.40 ± 0.98
**NIP-a**	Before incubation	883 ± 91	214 ± 29	7.39 ± 4.00	25.3 ± 10.9
	After incubation	748 ± 86	212 ± 78	2.11 ± 1.00	19.0 ± 5.6
**NIP-b**	Before incubation	384 ± 31	20 ± 13	2.30 ± 0.62	5.21 ± 1.33
	After incubation	419 ± 29	34 ± 20	4.05 ± 0.60	5.70 ± 1.16

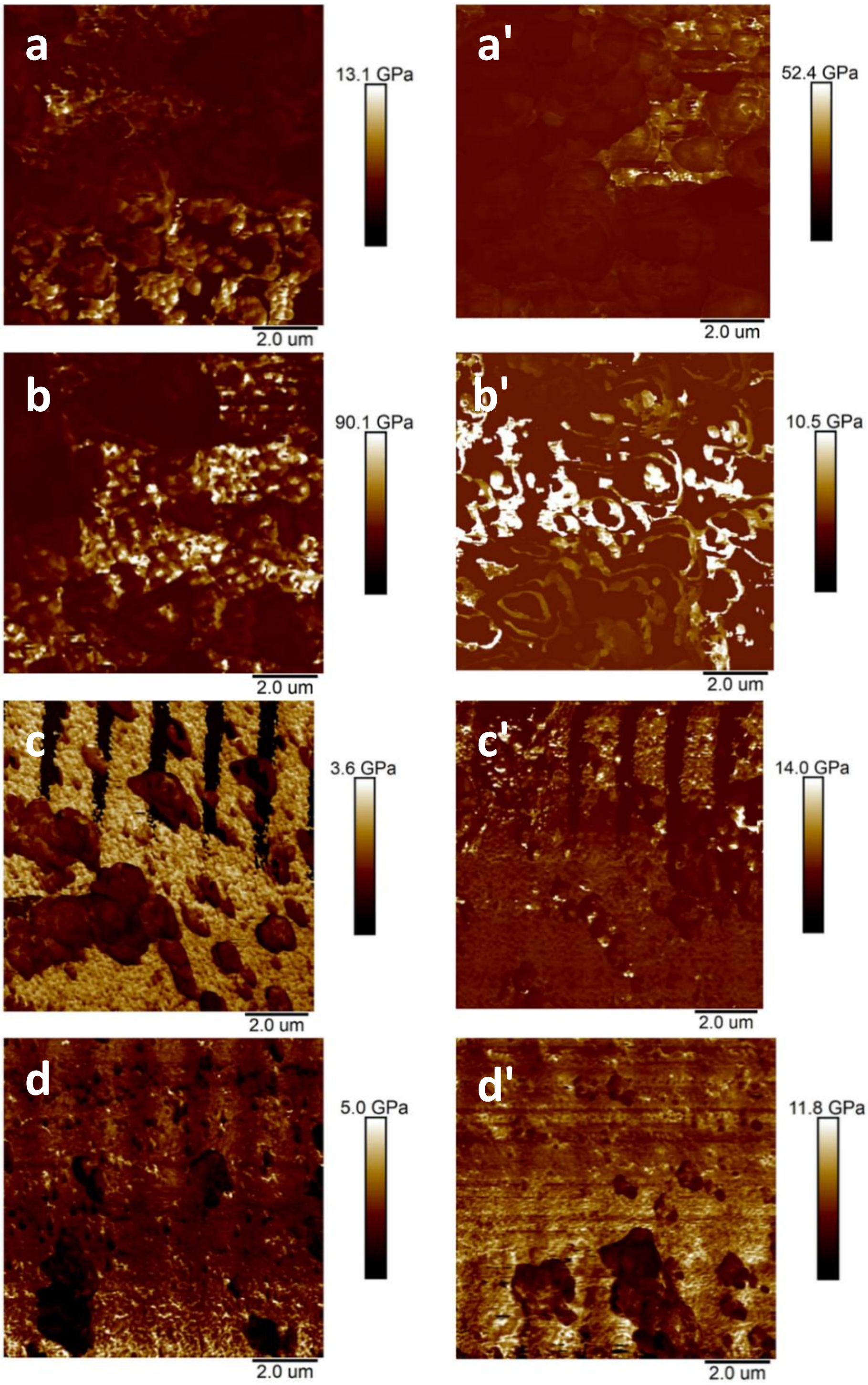

Figure S8. Young modulus maps recorded during AFM imaging of as prepared (**a**) **MIP-a**, (**b**) **MIP-b**, (**c**) **NIP-a**, and (**d**) **NIP-b** films as well as template-extracted (**a'**) **MIP-a**, (**b'**) **MIP-b**, and extraction solution incubated (**c'**) **NIP-a**, and (**d'**) **NIP-b** films. All films were deposited on Au-layered glass slides by potentiodynamic electropolymerization.

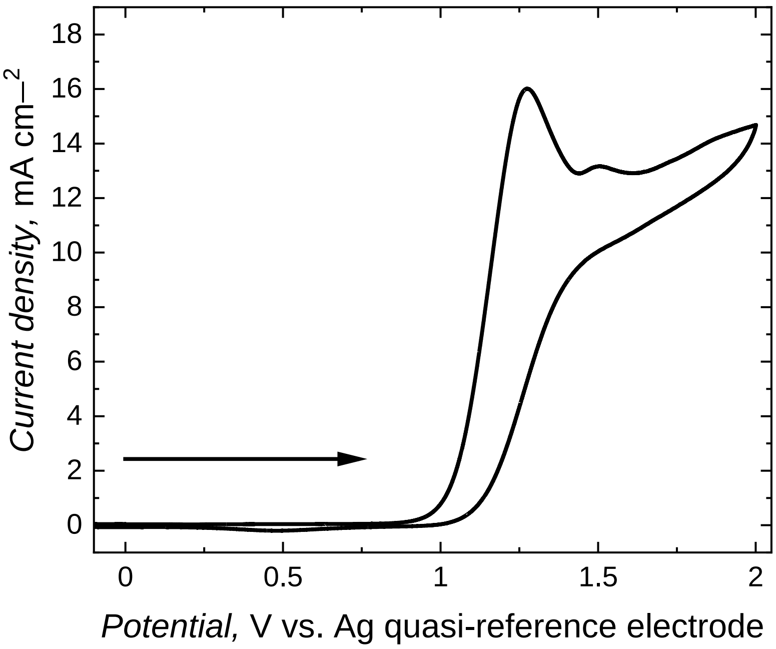

Figure S9. The cyclic voltammogram for 20 mM 2,4-dimethylphenol (**DMPh**) in 100 mM (TBA)ClO_4 in acetonitrile recorded at a Pt disk bare electrode at a scan rate of 100 mV s^{-1}.

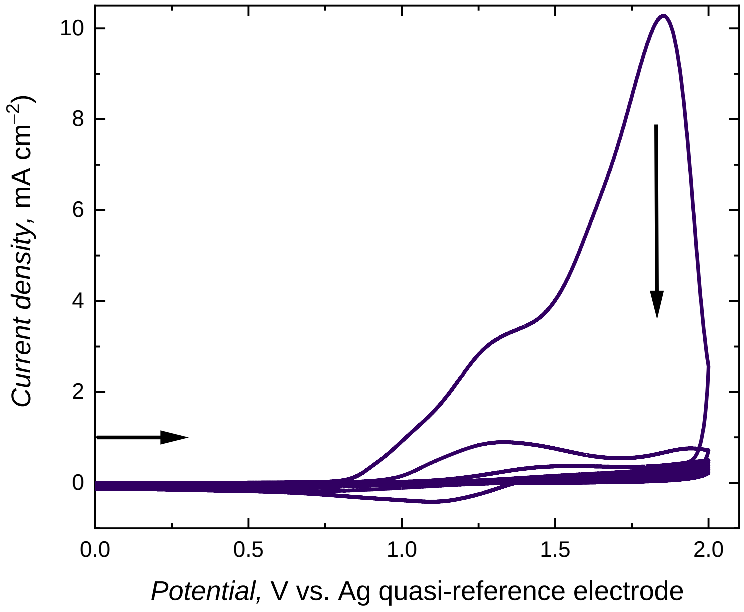

Figure S10. The ten-cycle CV curve of pre-treatment of the **MIP-a** film-coated Pt electrode in the acetonitrile : dichloromethane (9:1, *v/v*) solution of 100 mM (TBA)ClO_4.

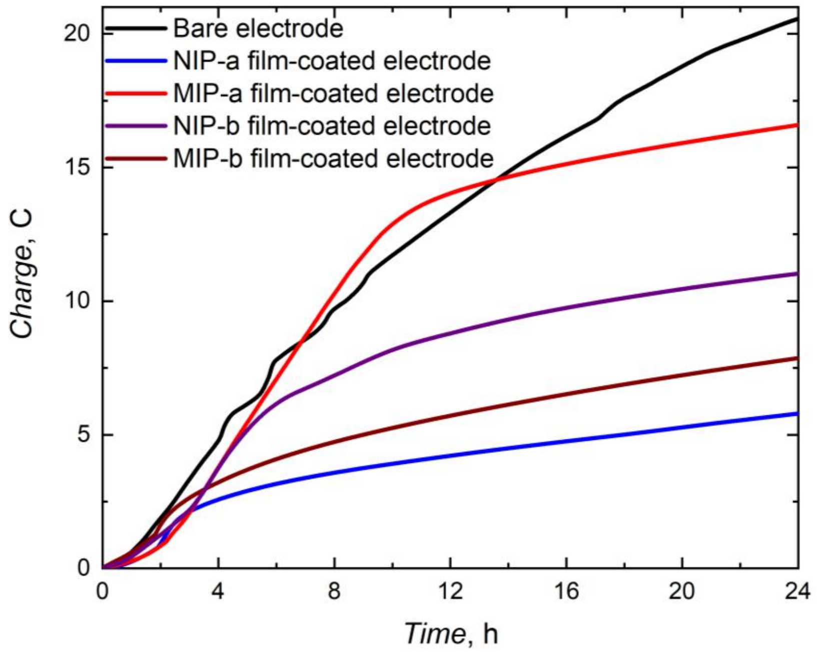

Figure S11. The charge passed as a function of electrosynthesis time during electro-oxidation of 20 mM **DMPh** at the bare, as well as **MIP-a**, **MIP-b**, as well as **NIP-a** and **NIP-b** film-coated 1.90-cm^2 active area Pt plate electrode at 1.20 V vs. Ag quasi-reference electrode in the acetonitrile : dichloromethane (9 : 1, *v/v*) solution of 100 mM (TBA)ClO_4.

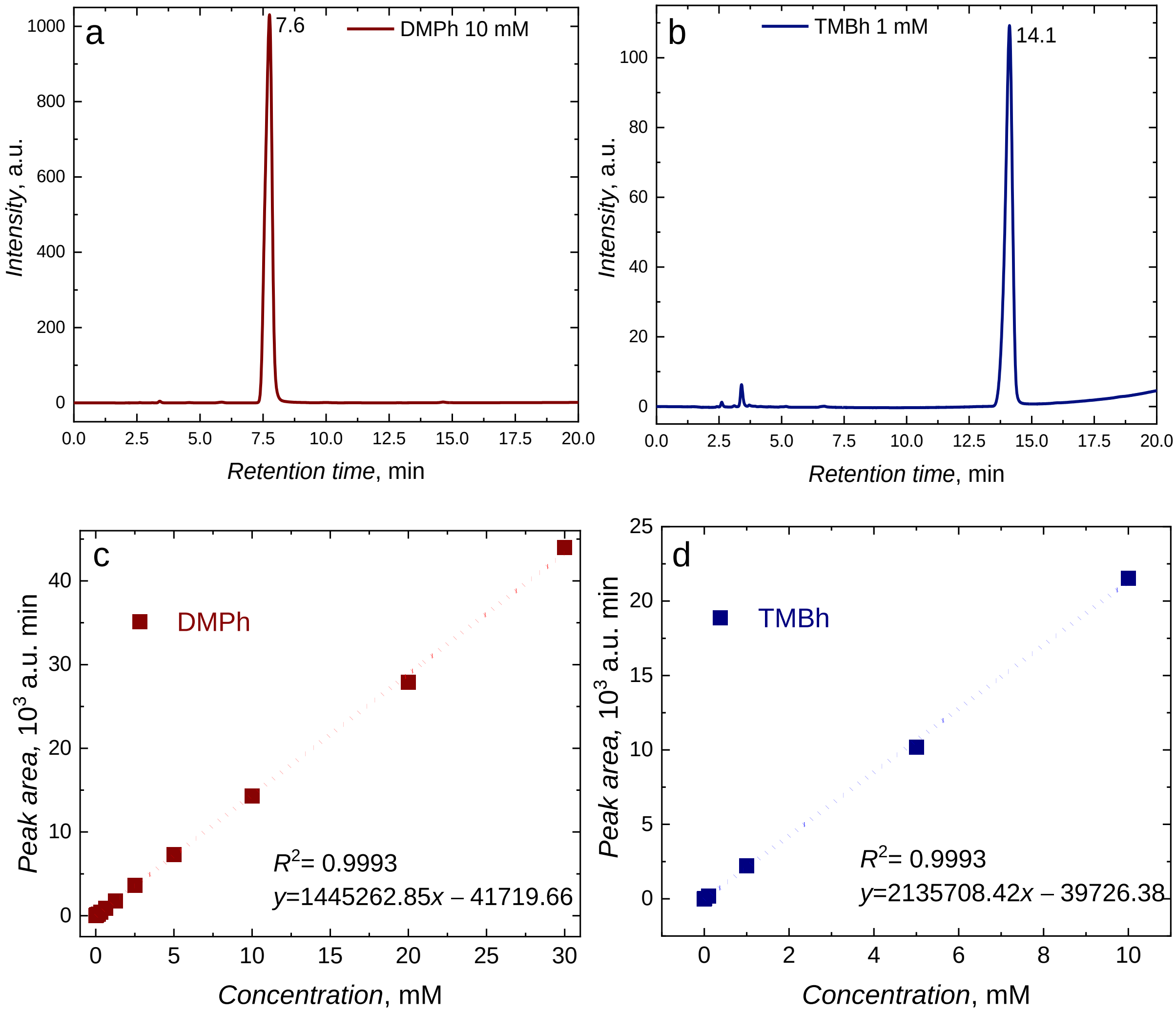

Figure S12. HPLC chromatograms at 280 nm for (**a**) the **DMPh** substrate and (**b**) the desired **TMBh** product. HPLC calibration plots for (**c**) the **DMPh** substrate and (**d**) the **TMBh** desired product. The compounds were analyzed using a Luna 5µm C18(2) 100 Å reversed-phase liquid chromatography column. A mobile phase composed of ultrapure water (Solvent A) and ACN (Solvent B) was used for gradient elution. A linear gradient was used from a 50 : 50 ratio (Solvent A : Solvent B, *v*/*v*) at 0 time to a 5 : 95 ratio at 20 minutes.

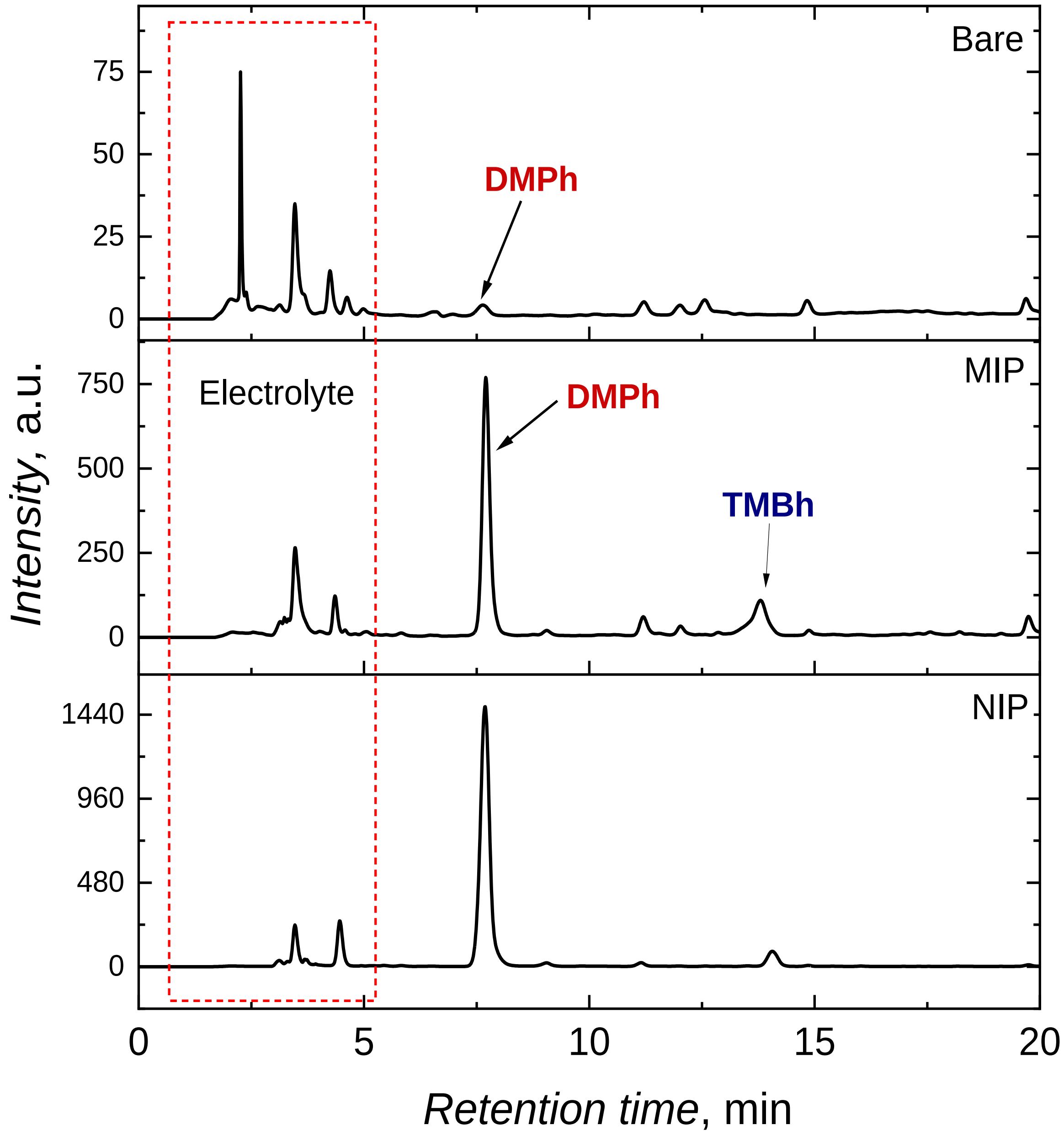

Figure S13. Exemplary HPLC chromatograms at 280 nm for fractions collected after 14-h electrosynthesis at the bare, as well as the **MIP-a** and **NIP-a** film-coated electrodes. The compounds were analyzed using a Luna 5µm C18(2) 100 Å reversed-phase liquid chromatography column. A mobile phase composed of ultrapure water (Solvent A) and ACN (Solvent B) was used for gradient elution. A linear gradient was used from a 50 : 50 ratio (Solvent A : Solvent B, *v*/*v*) at 0 time to a 5 : 95 ratio at 20 minutes.

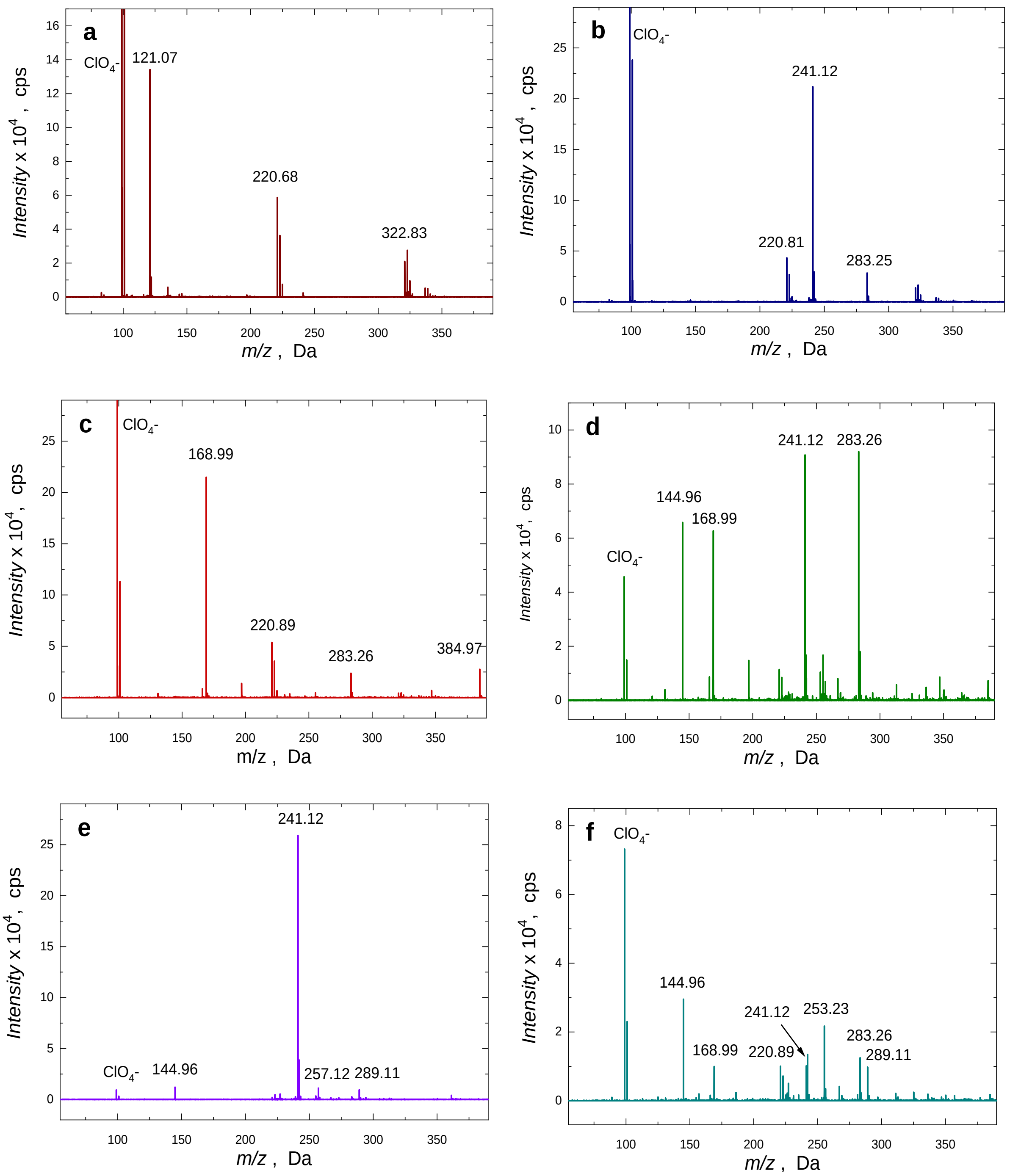

Figure S14. Mass spectra of reference compounds of (**a**) **DMPh** and (**b**) **TMBh**, as well as HPLC fractions of the reaction mixture collected at the retention time of (**c**) 6.7, (**d**) 11.0, (**e**) 14.9, and (**f**) 19.7 min. All spectra were acquired using the negative ion mode and the atmospheric pressure chemical ionization (APCI).

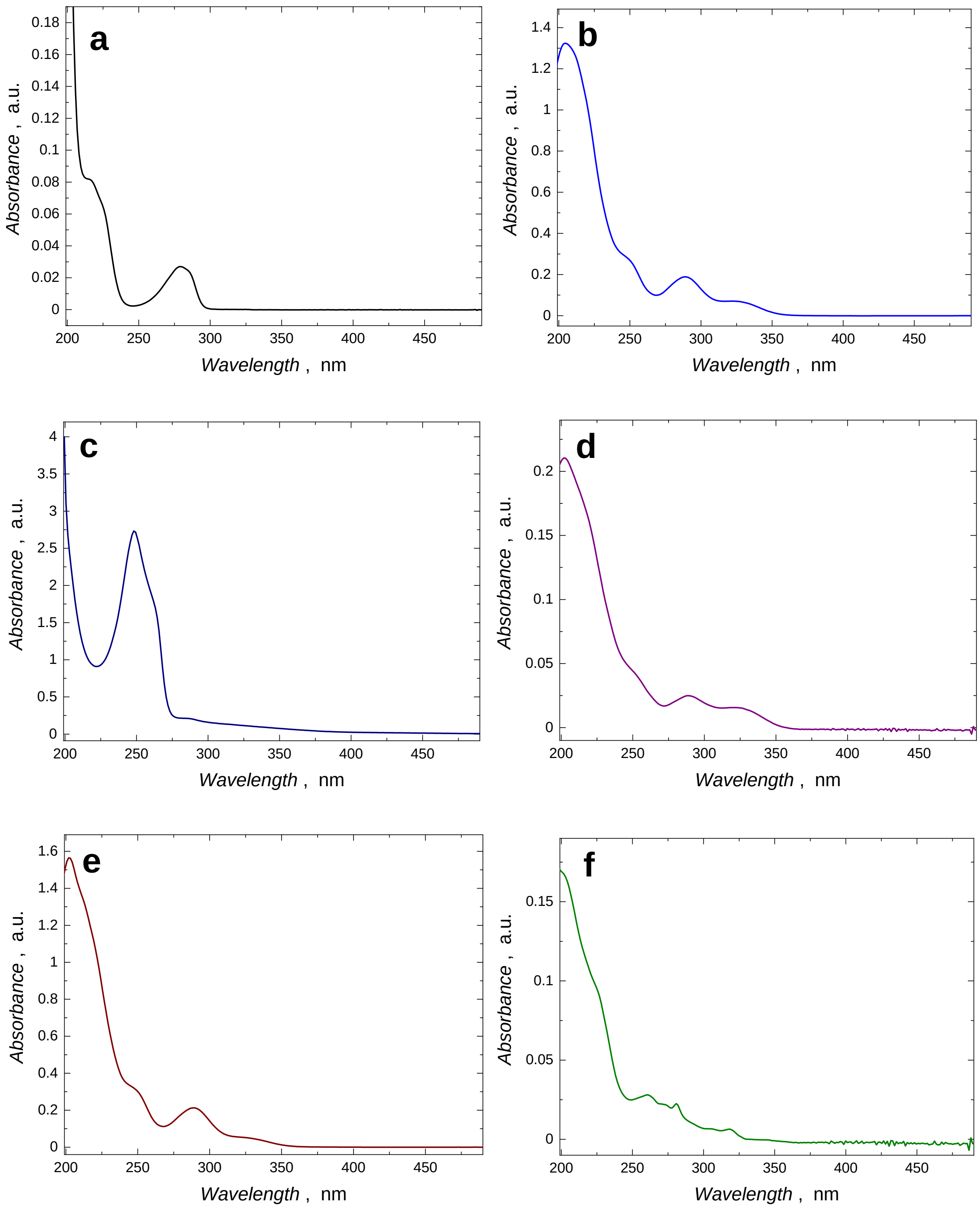

Figure S15. UV-vis spectra for (**a**) the **DMPh** substrate and (**b**) the desired **TMBh** product, as well as for HPLC fractions of the reaction mixture collected at the retention time of (**c**) 6.7, (**d**) 11.0, (**e**) 14.9, and (**f**) 19.6 min. All spectra were acquired using a diode-array UV-vis detector.

a **b**

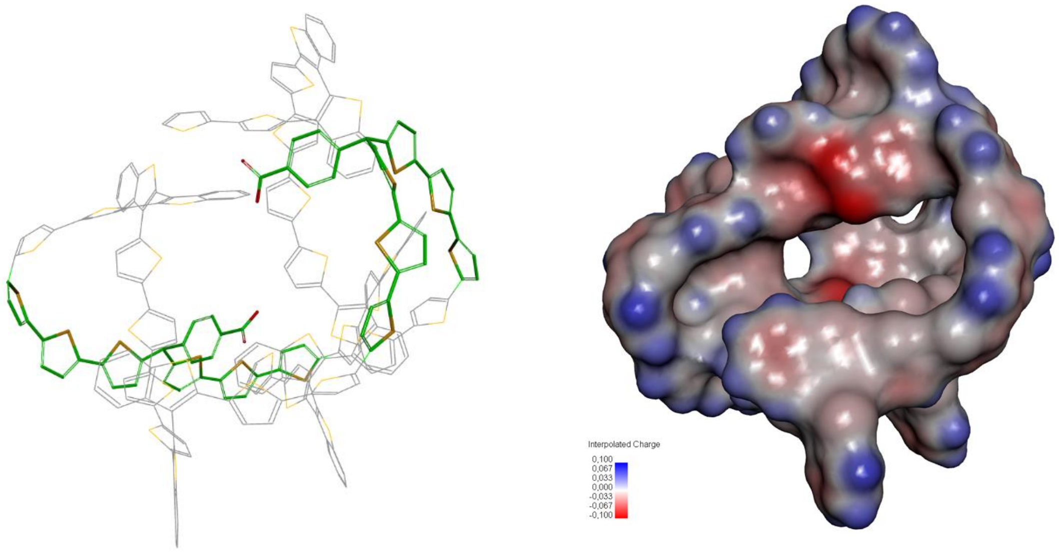

Figure S16. A computationally simulated structure of the molecular cavity imprinted in the **MIP**-**TMBh** film. (**a**) A skeleton model and (**b**) molecular electrostatic potential (MEP) surface, colored according to the interpolated charge (blue and red represent the positive and negative charge, respectively).

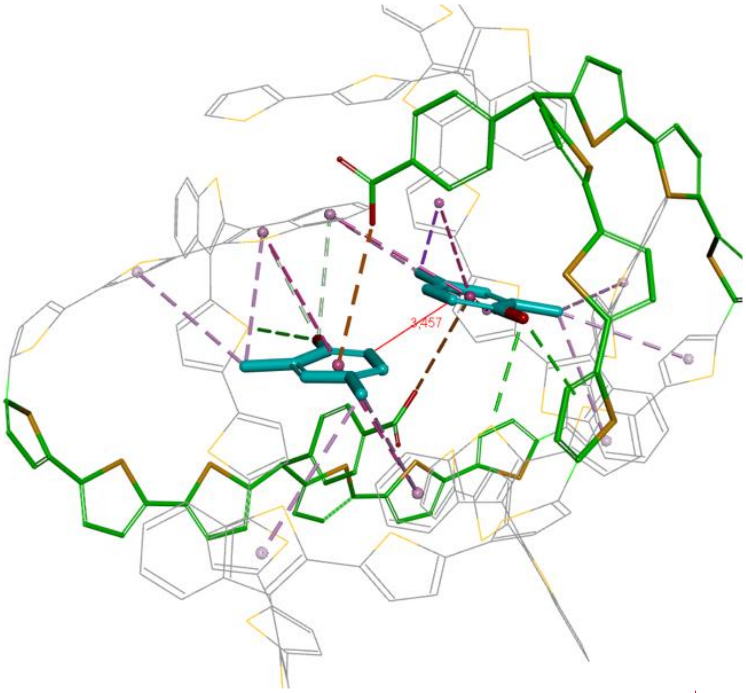

Figure S17. The computationally simulated interactions of two **DMPh**$^{•+}$ substrate radical cations, indicated with dash segments, in the skeleton model of the **MIP** cavity.
